# ERG phase separation attenuates cellular senescence

**DOI:** 10.1016/j.isci.2026.114678

**Published:** 2026-01-13

**Authors:** Lu Pu, Zhiliang Zuo, Hui Zheng, Rui Ou, Ru Gao, Zhaomin Deng, Xiaochu Wu, Chun Xiao, Meiling Ge, Lixing Zhou, Haoran Jin, Shaochong Qi, Fengjuan Hu, Jieli Chen, Hang Li, Yan Zhao, Birong Dong, Hao Jiang

**Affiliations:** 1 Laboratory for Aging and Cancer Research, National Clinical Research Center for Geriatrics, West China Hospital, Sichuan University, Chengdu 610041, Sichuan, China; 2 Laboratory for Aging and Cancer Research, Frontiers Science Center for Disease-related Molecular Network, West China Hospital, Sichuan University, Chengdu 610041, China; 3 The Center of Gerontology and Geriatrics, West China Hospital, Sichuan University, Chengdu 610041, China; 4 National Clinical Research Center of Geriatrics, West China Hospital, Sichuan University, Chengdu 610041, China; 5 Department of Cardiology, The Third People’s Hospital of Chengdu, The Affiliated Hospital of Southwest Jiaotong University, Cardiovascular Disease Research Institute of Chengdu, Chengdu 610041, China; 6 State Key Laboratory of Respiratory Disease, National Clinical Research Center for Respiratory Disease, National Center for Respiratory Medicine, Guangzhou Institute of Respiratory Health, the Fisrt Affiliated Hospital of Guangzhou Medical University, Guangzhou, Guangdong 510120, China; 7 School of Sports Medicine and Health, Chengdu Sport University, Chengdu 610041, China; 8 The People’s Hospital of Ya’an, Ya’an 625000, China; 9 The People’s Hospital of Wenjiang Chengdu, Chengdu 611130, China; 10 Department of Medical Genetics, State Key Laboratory of Biotherapy, West China Hospital, Sichuan University, Chengdu 610041, China; 11 Department of Gastroenterology and Hepatology, West China Hospital, Sichuan University, Chengdu 610041, China; 12 Sichuan University-University of Oxford Huaxi Joint Centre for Gastrointestinal Cancer, Frontiers Science Center for Disease-Related Molecular Network, West China Hospital, Sichuan University, Chengdu 610041, China; 13 The Quzhou Affiliated Hospital of Wenzhou Medical University, Quzhou People’s Hospital, Quzhou 324000, China; 14 Ziyang Zhonghua Rehabilitation Hospital, Ziyang Environmental Science and Technology Vocational College, Ziyang 618400, China; 15 Chengdu Eighth People’s Hospital, Geriatric Hospital of Chengdu Medical College, Chengdu 610081, China; 16 The Fifth People’s Hospital of Sichuan Province, Chengdu 610031, China

**Keywords:** medical biochemistry, chromosome organization, biophysical chemistry

## Abstract

Centenarians, individuals who reach extreme old age, provide a valuable model for understanding mechanisms associated with healthy aging. Using ATAC-seq and transcriptomic profiling of peripheral blood mononuclear cells from centenarians, we identified a distinct chromatin accessibility landscape linked to exceptional longevity. Integrative analysis highlighted the E-26 transformation-specific (ETS)-related transcription factor ERG as a longevity-associated regulator. Functional studies in human cells showed that ERG forms nuclear condensates through liquid-liquid phase separation, a property associated with altered chromatin organization and reduced expression of senescence-related genes, including CDKN2A. Consistent with these effects, ERG condensation was associated with attenuation of cellular senescence phenotypes. Together, these findings connect epigenomic features observed in centenarians with transcription factor biophysical properties and cellular aging control, highlighting phase separation as a regulatory layer that may contribute to cellular resilience during aging.

## Highlights

• Centenarian PBMCs exhibit distinct chromatin accessibility landscapes
• ERG emerges as a transcription factor associated with human longevity
• ERG phase separation represses CDKN2A transcription to limit cellular senescence

## Graphical abstract

**Figure:**
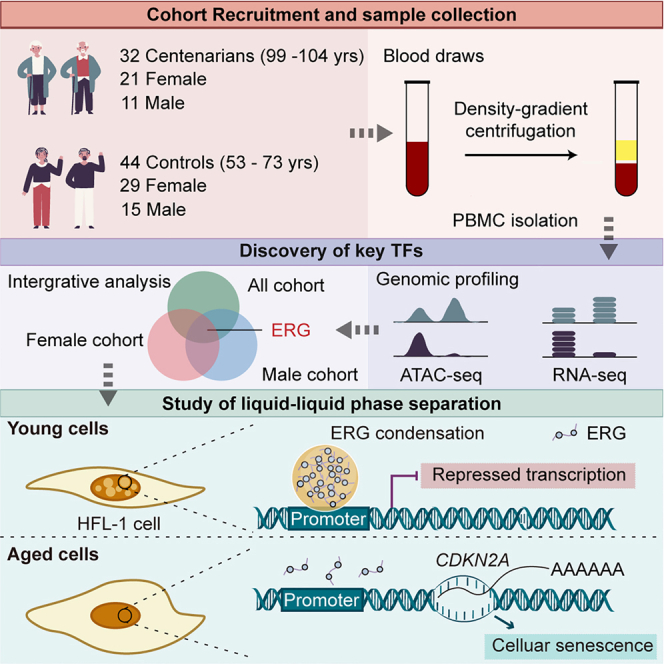

## Introduction

Aging is a complex and progressive process characterized by a gradual decline in physiological functions, leading to increased morbidity and mortality. Although advances in medical technology have significantly extended human lifespan, the growing proportion of older individuals poses challenges to healthcare systems due to the increasing burden of age-related diseases.^1^ Centenarians are individuals who have reached the age of 100 years or older and represent exceptional models of healthy aging, typically exhibiting delayed onset or absence of age-related disorders.^2^ Investigating the molecular mechanisms underlying their remarkable longevity may provide key insights into the biology of aging and identify potential targets involved in healthy lifespan regulation.

Human peripheral blood mononuclear cells (PBMCs) are widely used to study cellular senescence because they accumulate age-associated molecular changes, are easily accessible, and can be analyzed *ex vivo*.^3^^,^^4^^,^^5^^,^^6^^,^^7^ Such studies may reveal potential biomarkers relevant to aging and longevity. However, whether centenarians possess distinct chromatin accessibility landscapes in PBMCs that contribute to their extended lifespan remains unclear.

Liquid-liquid phase separation (LLPS) has emerged as a fundamental mechanism of intracellular organization, enabling dynamic compartmentalization that supports diverse physiological functions and contributes to various diseases.^8^^,^^9^^,^^10^^,^^11^ Increasing evidence also links LLPS to cellular senescence and aging. For example, BuGZ nuclear condensates regulate gut homeostasis and lifespan in *Drosophila* by modulating intestinal stem cell proliferation through m^6^A factors^12^; aberrant phase transitions of FUS RNA binding protein (FUS) drive hematopoietic stem cell aging by altering chromatin architecture^13^; and nuclear condensation of SGF29, a component of the Spt-Ada-Gcn5 acetyltransferase complex, accelerates cellular aging.^14^ Several transcription factors (TFs) have likewise been shown to form phase-separated condensates that facilitate long-range DNA interactions and coordinate gene expression programs. Notably, the TF EHF induces senescence without activating the senescence-associated secretory phenotype (SASP) in pancreatic ductal adenocarcinoma by forming condensates that repress telomerase reverse transcriptase and inflammatory cytokines.^15^ Despite these advances, whether TFs undergo LLPS to regulate longevity, and the mechanisms by which such condensates influence aging biology, remains largely unexplored.

Here, we use ATAC-seq to map the chromatin accessibility landscape of PBMCs from centenarians and uncover both conserved and sex-specific regulatory features associated with healthy aging. Among the key TFs identified, ERG emerged as a pivotal longevity-associated regulator. We demonstrate that ERG forms nuclear condensates through LLPS to modulate the expression of senescence-related genes, thereby linking TF phase separation to the maintenance of cellular youthfulness. Together, our findings provide new mechanistic insights into how ERG-mediated phase separation contributes to transcriptional regulation of repressing cell senescence.

## Results

### Global chromatin accessibility remodeling in PBMCs of centenarians

To investigate chromatin accessibility changes associated with exceptional longevity, we collected peripheral blood and isolated PBMCs from 76 community-dwelling healthy volunteers (50 women, 26 men), including 44 controls (age: 53–73 years; 29 women, 15 men) and 32 centenarians (age: 99–106 years; 21 women, 11 men) (Table S1). ATAC-seq profiles were successfully generated from 50 subjects (21 controls [13 women, 8 men] and 29 centenarians [20 women, 9 men]) to characterize genome-wide patterns of accessible chromatin (Table S1).

**Figure 1:**
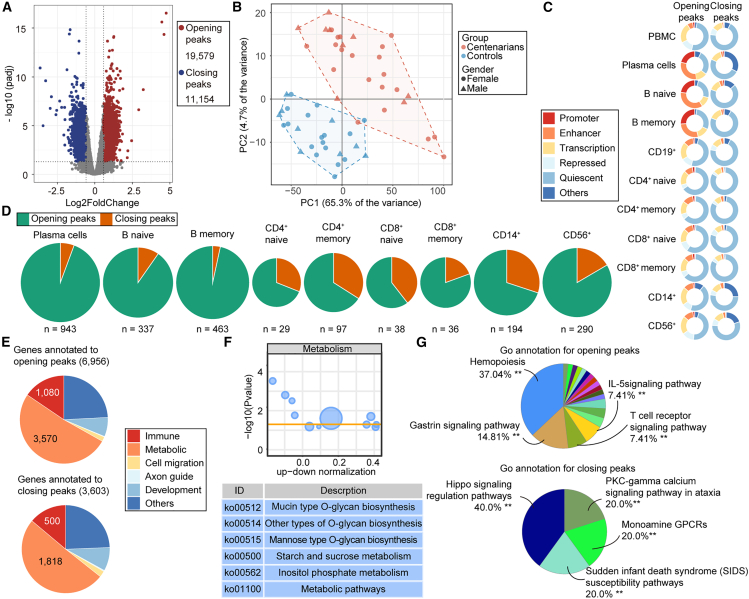
Global chromatin accessibility landscape in PBMCs of centenarians (A) Volcano plots of differentially accessible (DA) peaks between centenarians and controls. (B) Plot of principal-component analysis (PCA) based on DA peaks. (C) Functional annotations of opening and closing in centenarians using chromHMM states in PBMCs and cell subsets. (D) Distribution of DA peaks in centenarians among cell-specific loci from chromHMM states. (E) Major GO category annotations of genes associated with opening and closing peaks. (F) KEGG metabolic pathways of genes associated with DA peaks. (G) Significant GO terms associated with immune-related genes enriched among genes annotated to opening and closing peaks. Statistical significance was determined by ClueGO group enrichment analysis. Adjusted *p* values were calculated using Bonferroni-Holm step-down correction for multiple testing. ∗∗ indicates adjusted *p* < 0.01. See also Figure S1 and Table S1.

A total of 30,733 differentially accessible (DA) peaks (*p*adj < 0.05, absolute fold change >1.5) were identified between centenarians and controls. Among them, 19,579 peaks showed increased accessibility (opening peaks) and 11,154 showed decreased accessibility (closing peaks) in centenarians (Figures 1A and S1A). Principal-component analysis (PCA) and hierarchical clustering further revealed distinct accessibility profiles between centenarians and controls, with samples clearly segregating by age (Figures 1B and S1B–S1D).

Functional annotation of DA peaks using chromatin-state maps from the Roadmap Epigenomics Project^16^ revealed that most peaks were located within “quiescent” regions (i.e., loci without functional annotation) shared across immune cells (Figure 1C). However, a subset of opening peaks was enriched in regulatory elements (promoters and enhancers) of plasma cells, naive B cells, and memory B cells (Figure 1C). This pattern contrasts with a previous study^17^ in elderly individuals (>65 years), which reported that opening peaks were predominantly quiescent while closing peaks were enriched in regulatory loci, consistent with global chromatin deregulation during aging.^18^ These observations suggest that centenarians possess a distinctive and more functionally engaged chromatin accessibility profile compared to typical elderly subjects.

To further delineate cell type-specific patterns, we examined DA peaks active in individual immune cell subsets (Figure S1E). In centenarians, opening peaks were broadly distributed across all immune cell types (Figures 1D and S1F), reflecting a widespread gain of chromatin accessibility throughout the PBMC population. Interestingly, while monocyte-, NK-, and memory CD8^+^ T cell-specific opening peaks mirrored classical aging signatures,^17^ the accessibility patterns of naive T cells and memory CD4^+^ T cells deviated from age-associated trends,^17^ indicating centenarian-specific remodeling (Figures 1D and S1F). Moreover, B cell-specific loci were disproportionately affected, with 902 plasma cell, 458 memory B cell, and 307 naive B cell peaks showing significant accessibility gains (Figures 1D and S1F). These results highlight an epigenomic signature of centenarians characterized by a global yet selective remodeling of chromatin accessibility across immune lineages and most prominently in B cells.

DA peaks were annotated to genes with HOMER,^19^ linking 9,217 genes to opening peaks and 5,427 to closing peaks. Gene Ontology (GO) analysis revealed that metabolic processes, rather than immune functions, represented the largest category affected by chromatin accessibility changes: 3,570 metabolic genes were linked to opening peaks and 1,818 to closing peaks, while 1,080 immune-related genes were associated with opening peaks and 500 with closing peaks (Figure 1E). These results align with previous studies emphasizing the tightly interconnected nature of immune-metabolic regulation, a coevolved system that is essential for maintaining physiological homeostasis.^20^^,^^21^^,^^22^

Pathway analysis using the Kyoto Encyclopedia of Genes and Genomes (KEGG) indicated that DA peak-associated genes were predominantly enriched in pathways related to human diseases and organismal systems (Figure S1G). Metabolic pathways such as mucin-type *O*-glycan biosynthesis, other *O*-glycan biosynthesis, and mannose-type *O*-glycan biosynthesis (Figure 1F) were significantly affected. These pathways have been reported to be involved in hyperplastic pathologies and degenerative pathologies.^23^ In addition, starch and sucrose metabolism, previously linked to longevity,^24^ was also enriched, suggesting potential contributions of these chromatin changes to centenarian physiology.

ClueGO^25^ enrichment further revealed that opening peaks were significantly associated with genes involved in hematopoiesis-related GO terms (*n* = 444) and the gastrin signaling pathway (*n* = 66) (Figures 1G and S1H). The enrichment of hematopoietic pathways suggests preservation of blood cell production capacity in centenarians, whereas gastrin pathway activation may have mixed effects, as elevated gastrin levels in the elderly are associated with hypergastrinemia and an increased risk of gastric neoplasms.^26^

Conversely, closing peaks were enriched for genes involved in the Hippo signaling pathway (*n* = 33) (Figures 1G and S1H), a conserved network essential for tissue regeneration and cellular homeostasis.^27^ Its reduced activity in centenarians may reflect age-related attenuation of regenerative potential, balanced by other mechanisms supporting longevity.

Collectively, these results reveal both aging-associated and centenarian-specific chromatin accessibility signatures, characterized by coordinated remodeling across immune and metabolic genes that may contribute to the maintenance of physiological resilience at extreme old age.

### A transcriptomic and chromatin accessibility signature of immune resilience in centenarians

**Figure 2:**
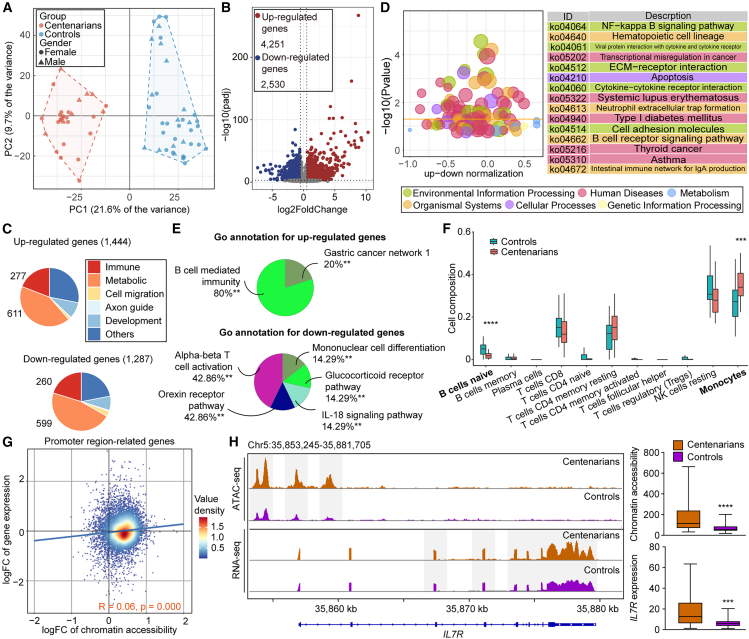
Limited transcriptional consequences of altered chromatin accessibility in centenarians (A) PCA plot of genes expression in PBMCs of centenarians and controls. (B) Volcano plots of differentially expressed (DE) genes between centenarians and controls. (C) Major GO category annotations of genes associated with up- and down-regulated genes. (D) KEGG pathways of DE genes. (E) Significant GO terms associated with immune-related genes enriched up- and down-regulated genes. Statistical significance was determined by ClueGO group enrichment analysis. Adjusted *p* values were calculated using Bonferroni-Holm step-down correction for multiple testing. ∗∗ indicates adjusted *p* < 0.01. (F) Proportions of immune cell types relative to total immune cells were estimated using CIBERSORTx. Group differences between centenarians and controls were assessed using a two-sided Wilcoxon rank-sum test. ∗*p* < 0.05; ∗∗*p* < 0.01; ∗∗∗*p* < 0.001; ∗∗∗∗*p* < 0.0001. (G) Correlation between chromatin accessibility and gene expression at promoters in centenarians. The correlation was assessed using a linear regression model (lm). *R* = 0.06, *p* < 0.0001. Each point represents one gene, and the color density indicates the number of overlapping points. (H) Average chromatin accessibility and transcriptional profiles around the IL7R locus (left), and the chromatin accessibility and gene expression levels of IL7R (right) in centenarians and controls. Statistical significance between groups was assessed using a two-sided Mann-Whitney test. ∗*p* < 0.05; ∗∗*p* < 0.01; ∗∗∗*p* < 0.001; ∗∗∗∗*p* < 0.0001. See also Figure S2 and Table S1.

We generated RNA-seq profiles of PBMCs from 63 subjects (35 controls [24 women, 11 men] and 28 centenarians [19 women, 9 men]) (Table S1) to investigate transcriptional changes accompanying the altered chromatin landscape in centenarians. PCA revealed that PBMCs segregated distinctly by age along the first principal component (Figure 2A). Differential expression analysis identified 6,781 differential expressed (DE) genes (4,251 upregulated and 2,530 downregulated; *p*adj < 0.05, absolute fold change >1.5) between centenarians and controls (Figure 2B). Consistent with genes linked to DA peaks, the majority of DE genes were metabolism-related rather than immune-related. GO analysis revealed 611 upregulated and 599 downregulated metabolic genes, while 277 upregulated and 260 downregulated genes were immune-related (Figure 2C).

The KEGG pathway classification showed that most DE genes were enriched in human disease- and organismal system-related categories, particularly cancer- and immune-related pathways (Figure 2D). However, only one metabolic pathway, one carbon pool by folate (*p* = 0.059), which has previously been implicated in aging,^28^ approached significance (Figure S2A). ClueGO enrichment further demonstrated that upregulated genes were enriched in B cell-mediated immunity (*n* = 35 genes) and gastric cancer network 1 (*n* = 9 genes) (Figures 2E and S2B), while downregulated genes were associated with α-β T cell activation (*n* = 30 genes) and the orexin receptor signaling pathway (*n* = 27 genes) (Figures 2E and S2B).

To examine whether cell composition shifts contributed to these transcriptional changes, we deconvoluted PBMC cell types using CIBERSORTx.^29^ The estimated proportion of naive B cells significantly decreased in centenarians, whereas monocytes increased (Figure 2F). These results are consistent with previous reports that the age-related increase in naive B cells does not persist in extreme longevity, while monocyte expansion reflects an adaptive feature of healthy aging.^30^^,^^31^^,^^32^^,^^33^

To determine whether chromatin accessibility changes correlate with transcriptional regulation, we integrated ATAC-seq and RNA-seq data using gene annotations derived from both HOMER^19^ and GREAT.^34^ Genome-wide analyses revealed a weak but statistically significant correlation between chromatin accessibility and gene expression across all genes, as well as within DE and DA gene subsets (Figures 2G and S2C). These findings indicate that the transcriptional regulation in centenarians involves multiple layers of control, and changes in chromatin accessibility might somehow contribute to shaping the overall gene expression landscape.

Notably, *IL7R* is a key regulator of lymphocyte development and immune homeostasis,^35^ and previous studies have identified that the loss of chromatin accessibility around locus is associated with age-related decrease in IL7R expression.^17^^,^^36^ In centenarians, *IL7R* exhibited increased promoter accessibility accompanied by elevated mRNA expression (Figure 2H). Additional genes within the *IL7* signaling cascade, including *IL7*, *PTK2B*, *STAT1*, and *GSK3B*, also displayed chromatin opening (Figure S2D), suggesting that the decline in *IL7*-mediated signaling typically observed with aging may be attenuated or reversed in centenarians.

### Shared and sex-specific chromatin and transcriptional signatures in centenarians

**Figure 3:**
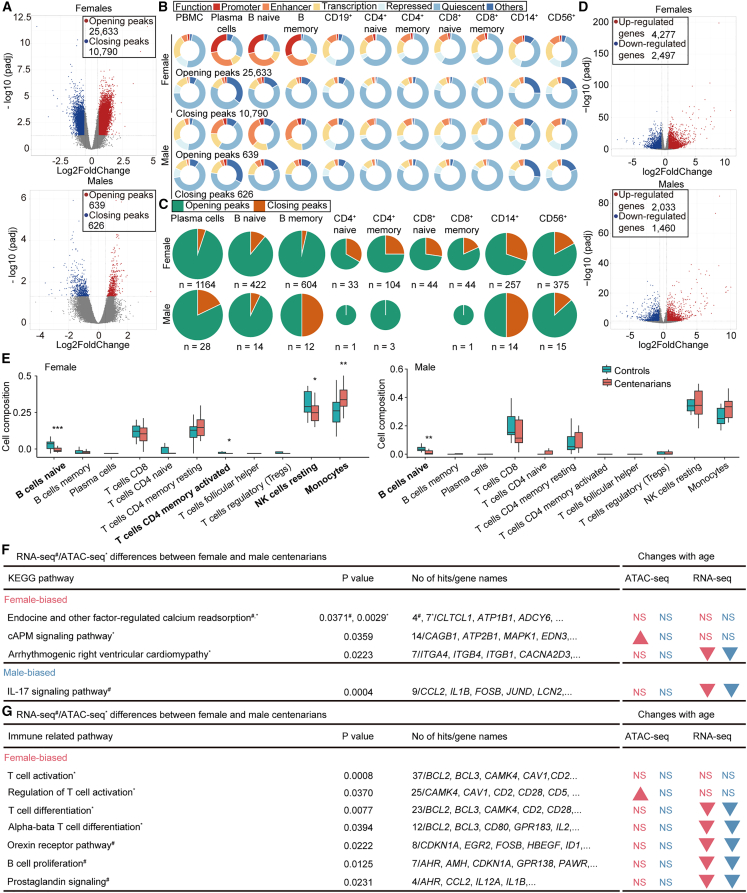
Sex-dependent chromatin remodeling and pathways activity in extreme aging (A) Volcano plots of DA peaks between female/male centenarians and controls. (B) Functional annotations of opening and closing in female/male centenarians using chromHMM states in PBMCs and cell subsets. (C) Distribution of DA peaks in female/male centenarians among cell-specific loci from chromHMM states. (D) Volcano plots of DE genes between female/male centenarians and controls. (E) Proportion of cell types as a total of all immune cells in female/male centenarians and controls. Group differences between centenarians and controls were assessed using a two-sided Wilcoxon rank-sum test. ∗*p* < 0.05; ∗∗*p* < 0.01; ∗∗∗*p* < 0.001; ∗∗∗∗*p* < 0.0001. (F and G) Selected KEGG (F) and immune -related (G) pathway enrichments for male-biased or female-biased genes/loci (left). For KEGG enrichment (F), statistical significance was assessed using a hypergeometric test with Benjamini-Hochberg correction for multiple testing. For ClueGO enrichment (G), group enrichment analysis was performed with Bonferroni-Holm step-down correction. Arrows indicate whether the same pathway/module was significantly associated with differences between female or male centenarians and controls. Arrow directions represent the direction of change, with red for women, blue for men, and NS, not significant. See also Figure S3.

Aging exerts distinct effects on the immune systems of men and women,^37^^,^^38^ yet how sex modulates the epigenome and transcriptome in extreme longevity remains underexplored. To address this, we compared sex-specific chromatin accessibility between centenarians and controls. Female centenarians exhibited 36,423 DA peaks (25,633 opening and 10,790 closing), whereas males centenarians showed only 1,265 (639 opening and 626 closing) compared to female and male controls, respectively (Figure 3A), indicating a broader chromatin remodeling landscape in women.

Closing peaks were largely quiescent loci across immune cell types in both sexes, whereas opening peaks in female B cells were mainly located in promoters and enhancers, and in males predominantly in enhancers (Figure 3B). The age-related enhancer gain observed in monocytes and NK cells^17^ was not retained in centenarians, suggesting a centenarian-specific redistribution of chromatin accessibility. In female centenarians, opening peaks were enriched across all immune cell types (Figure 3C). Compared to elderly women, their naive T and CD4^+^ memory cells maintained a more youthful chromatin state. Male centenarians exhibited opening peaks primarily in plasma, naive B, and NK cells, with few changes in T cells—likely due to a smaller sample size. Despite reported sex differences in B cell aging,^17^ both male and female centenarians showed increased accessibility at B cell-specific loci (Figure 3C), indicating shared epigenomic signatures of immune maintenance.

Genes linked to DA peaks (11,323 opening and 5,450 closing in females; 588 opening and 557 closing in males) were predominantly enriched in metabolic and immune-related pathways, with substantial overlap between sexes (Figures S3A and S3B).

RNA-seq analysis identified 4,277 upregulated and 2,497 downregulated genes in female centenarians and 2,030 upregulated and 1,460 downregulated genes in males (Figure 3D). Differentially expressed genes were likewise dominated by metabolic and immune functions, and 2,414 genes were shared across sexes (Figures S3C and S3D).

Deconvolution of PBMCs revealed decreased naive B, CD4^+^ memory T, and NK cell fractions and increased monocytes in female centenarians, whereas only naive B cells decreased in males (Figure 3E). Chromatin accessibility and gene expression were weakly but significantly correlated in both sexes (Figure S3E).

Direct comparison between sexes revealed minimal differences among controls but substantial divergence in centenarians, characterized by 979 DA peaks (*p* < 0.001, absolute fold change >1.5) and 812 DE genes (*p*adj <0.05, absolute fold change >1.5) (Figure S3F). Notably, these sex-associated chromatin and transcriptional changes were significantly positive correlated across ATAC-seq (R = 0.6, *p* < 0.001) and RNA-seq (R = 0.74, *p* < 0.001) datasets (Figure S3G).

Both DA peaks and DE genes associated with endocrine- and factor-regulated calcium reabsorption were more active in PBMCs of female centenarians compared with male centenarians (Figure 3F, left table). This is consistent with reported age-dependent sex differences in serum calcium levels, which become higher in women than in men after age 45.^39^ Epigenomic enrichment further showed that female-biased peaks were enriched for the cAMP signaling pathway, with significantly higher activity in female centenarians than in sex-matched controls (Figure 3F, left and right tables). As elevated cAMP can suppress pro-inflammatory mediators and enhance anti-inflammatory responses across multiple immune cell types,^40^ these results suggest that female centenarians may possess stronger anti-inflammatory capacity compared with controls or male centenarians.

ATAC-seq also revealed increased activity of arrhythmogenic tight ventricular cardiomyocytes in female centenarians relative to males (Figure 3F, left table). Although this pathway was downregulated with age in both sexes (Figure 3F, right table), the sex-specific difference among centenarians suggests a more pronounced age-related decline in cardiac-associated signaling in men. In contrast, IL-17 signaling activity was higher in male centenarians (Figure 3F, left table). Gene expression of IL-17 pathway components decreased with age in both sexes (Figure 3F, right table), but the greater reduction in females indicates a more substantial dampening of inflammatory signaling in women during exceptional aging.

ClueGO analysis showed that female-biased peaks were enriched for T cell differentiation and activation pathways (Figure 3G, left table). Although these pathways exhibited minimal or negative transcriptional changes with age in both sexes (Figure 3G, right table), their selective chromatin accessibility gains in female centenarians suggest preservation of T cell regulatory potential despite overall transcriptional decline, which is consistent with prior evidence that females generally mount stronger adaptive immune responses than males.^41^^,^^42^^,^^43^

Moreover, genes involved in orexin receptor activity,^44^ B cell proliferation,^45^ and prostaglandin signaling^46^ showed age-related downregulation in both sexes, with a more pronounced reduction in females (Figure 3G), reflecting a coordinated suppression of pro-inflammatory processes associated with longevity.

In summary, centenarians display both shared and sex-specific epigenomic and transcriptomic features. Female centenarians exhibit broader chromatin remodeling and enhanced anti-inflammatory signatures, whereas males show more constrained yet coordinated transcriptional adaptations. Together, these differences collectively highlight immune resilience as a hallmark of extreme longevity.

#### ERG as a longevity-associated TF shaping the centenarian chromatin landscape

**Figure 4:**
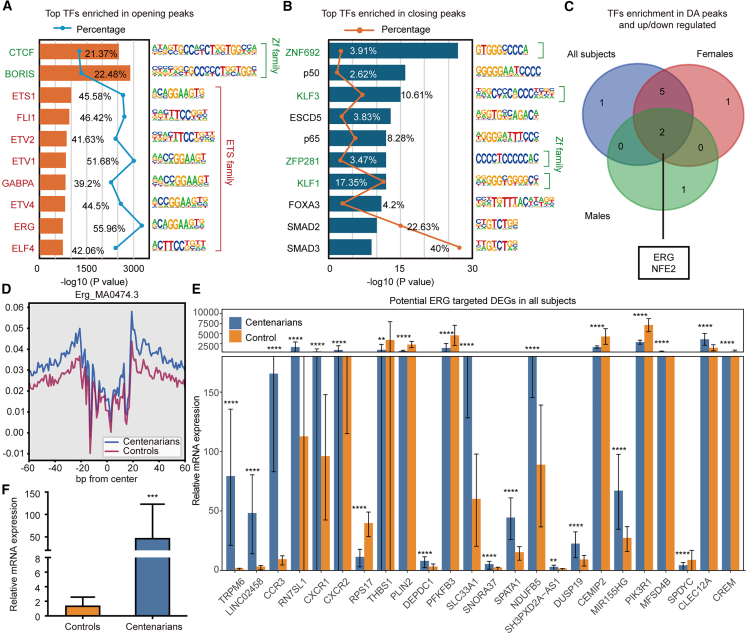
ERG emerges as a longevity-associated transcription factor (TF) in centenarians (A and B) Top enriched known TF motifs of opening (A) and closing (B) peaks. (C) Venn diagram showing overlap of the corresponding TFs for each motif enriched in DA peaks and DE genes from all, female and male, centenarians. (D) ERG motif footprint. (E) Bar charts showing the expression level of potential ERG target genes. (F) RT-qPCR results of ERG expression in PBMCs from centenarians and controls. Data are presented as mean ± SD. Statistical significance between groups was assessed using a two-sided Mann-Whitney test. ∗*p* < 0.05; ∗∗*p* < 0.01; ∗∗∗*p* < 0.001; ∗∗∗∗*p* < 0.0001. See also Figure S4 and Table S1.

To identify potential regulators of the epigenomic remodeling observed in centenarians, we performed motif enrichment analysis on loci with altered chromatin accessibility. Analyses were conducted in both overall and sex-stratified cohorts to reveal shared and sex-specific regulators of longevity. Among regions that gained accessibility, motifs for zinc finger and E-26 transformation-specific (ETS) family TFs, including CTCF, ETS1, and ERG, were significantly enriched (Figure 4A). CTCF, a zinc finger TF critical for genome organization and transcriptional insulation, is known to decline during replicative senescence and inflammation^47^^,^^48^^,^^49^; thus, its enrichment in centenarians suggests a role in maintaining chromatin stability during healthy aging.

ETS1 motifs, typically enriched in loci that lose accessibility with age, particularly in CD8^+^ T cells,^50^ were regained in centenarians, implying a chromatin landscape more similar to that of younger individuals. ETS1 also regulates ribosomal protein gene expression,^51^ consistent with decreased ribosomal activity observed in our data. Subgroup analysis of opening peaks showed largely consistent TF motif enrichment between sexes, with over 85% overlap (Figure S4A). Closing peaks were mainly enriched for zinc finger TFs, primarily driven by females, and SMAD2 motifs were enriched in closing peaks of both sexes, consistent with its role in regulating growth, differentiation, and apoptosis (Figures 4B and S4B).^52^^,^^53^

To identify TFs mediating chromatin and transcriptional changes, motif-enriched TFs were intersected with DE genes. In the overall cohort, six upregulated and two downregulated TFs were associated with opening and closing peaks, respectively (Figure S4C). Sex-stratified analysis showed seven upregulated and one downregulated in females, and three upregulated in males (Figure S4C). ERG and NFE2 were consistently upregulated and enriched in regions gaining accessibility across all groups (Figures 4C and S4C–S4E). TOBIAS footprinting^54^ confirmed increased occupancy of these motifs in centenarians compared to controls (Figures 4D and S4F).

Potential ERG target genes were identified based on the presence of ERG motifs within DA regions linked to nearby genes. RNA-seq profiles revealed corresponding expression alterations in these genes (Figure 4E), providing supportive, though not definitive, evidence for ERG-mediated regulation. In contrast, predicted NFE2 targets showed no significant expression changes (Figure S4G). Consistently, RT-qPCR analysis confirmed elevated ERG expression in PBMCs of centenarians (Figure 4F; Table S1).

Motif analysis of sex-biased accessible regions further revealed preferential enrichment of ERG motifs in female-biased peaks, suggesting stronger or more sustained ERG-associated regulatory activity in female centenarians (Figure S4H). Among the putative ERG targets, MB21D2^55^ and N4BP3,^56^ both implicated in antiviral immune responses, displayed sex-dependent expression differences between female and male centenarians (Figure S4I). These findings suggest that ERG may contribute to sex-specific modulation of antiviral immunity during extreme aging.

Together, these findings identify ERG as a longevity-associated TF that may help preserve youthful chromatin accessibility and immune regulatory capacity in centenarians, motivating subsequent investigations into functional of ERG contributions to cellular senescence.

### ERG undergoes liquid-liquid phase separation in cells and *in vitro*

Recent studies have demonstrated that many TFs undergo LLPS, forming membrane-less condensates that compartmentalize biochemical reactions and modulate key cellular processes.^57^^,^^58^ We therefore sought to determine whether ERG could exhibit similar LLPS behavior and contribute to cellular regulation during aging.

To address this, we employed replicative-senescent human embryonic lung fibroblasts (HFL-1)^59^ and HEK293T cells. Droplet digital PCR revealed that endogenous ERG expression was extremely low in both cell types (23 and 64 copies per microliter in HFL-1 and HEK293T, respectively; Figure S5A). Considering that ERG expression was nearly undetectable in PBMCs from control individuals but markedly elevated in centenarians, HFL-1 fibroblasts provided an appropriate system for ERG overexpression and controlled reconstitution of LLPS to examine its role in cellular senescence.

Immunofluorescence staining of endogenous ERG in HEK293T cells revealed discrete nuclear puncta, which rapidly dissolved upon treatment with 1,6-hexanediol (1,6-HD) and reappeared after its removal (Figure S5B), consistent with liquid-like condensates.^60^ ERG knockdown substantially reduced the number of nuclear puncta, confirming staining specificity (Figures S5C and S5D). We also evaluated ERG condensates in a DNA damage-induced senescence model using bleomycin (BLM)-treated^61^ HEK293T cells. Following BLM exposure, ERG expression markedly decreased, whereas CDKN2A (p16) levels increased (Figure S5E), suggesting that ERG acts as a negative regulator of senescence. Moreover, the accompanying reduction in nuclear puncta of BLM-treated HEK293T (Figure S5F) further supported the specificity of the observed condensates.

**Figure 5:**
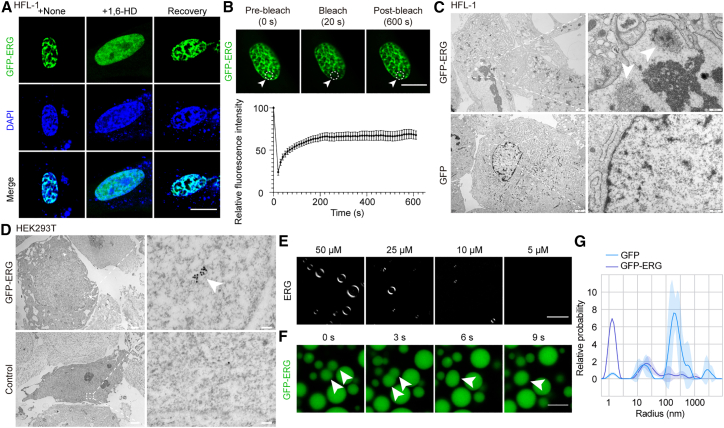
ERG underwent liquid-liquid phase separation in HFL-1 and HEK293T cells and *in vitro* (A) Representative images of GFP-ERG condensation in HFL-1 cells. The incubated and wash-out of 1,6-HD destroy and re-formation of the condensation in the nuclei. Scale bars, 10 μm (B) FRAP of GFP-ERG condensates in HFL-1 cells. The photobleached region is indicated by the white circle and arrow (upper panel). Quantification of fluorescence recovery over time from 15 individual cells is shown (lower panel). Data are presented as mean ± SEM. Scale bars, 10 μm (C) TEM image of HFL-1 cells overexpressing GFP-ERG or GFP. The condensation formed by GFP-ERG is indicated by white box and arrow. Scale bars, 2 μm (left); 500 nm (right). (D) Immunogold EM analysis of HEK293T cells overexpression of GFP-ERG and the negative control. Scale bars, 2 μm (left); 100 nm (right). (E) Droplet formation by different concentrations of ERG protein. Scale bars, 5 μm (F) Fusion of GFP-ERG droplets. Scale bars, 5 μm (G) Radius distribution of GFP-TFAP2β and GFP protein droplets measured by DLS. See also Figure S5.

In cells overexpressing GFP-ERG, irregular nuclear condensates^62^ were observed, exhibiting similar 1,6-HD sensitivity (Figures 5A and S5G). Fluorescence recovery after photo bleaching (FRAP) analysis demonstrated that GFP-ERG droplets were highly dynamic, with over 60% fluorescence recovery within 10 min in both cell types (Figures 5B and S5H). TEM revealed electron-dense structures consistent with liquid condensates in HFL-1 and HEK293T cells (Figures 5C and S5I), and immunogold labeling confirmed these GFP-ERG-enriched structures in HEK293T nuclei (Figure 5D).

*In vitro*, purified ERG protein spontaneously formed concentration-dependent droplets whose formation was modulated by pH and salt concentration (Figures 5E, S5J, and S5K). Similarly, the purified GFP-ERG exhibited fusion behaviors, a characteristic of LLPS (Figure 5F).^63^ The condensation dynamics of the purified GFP-ERG were further confirmed *in vitro via* FRAP assays (Figure S5L). However, the recovery *in vitro* was slower than that in cells, suggesting additional nuclear components may facilitate droplet fluidity *in vivo*. Moreover, dynamic light scattering (DLS) further confirmed that GFP-ERG formed droplets consistent with LLPS behavior (Figure 5G).

Together, these findings demonstrate that ERG undergoes LLPS both in living cells and *in vitro*, supporting its role in organizing nuclear architecture and potentially regulating gene expression and cellular function during aging.

#### ERG phase separation mitigates cellular senescence

**Figure 6:**
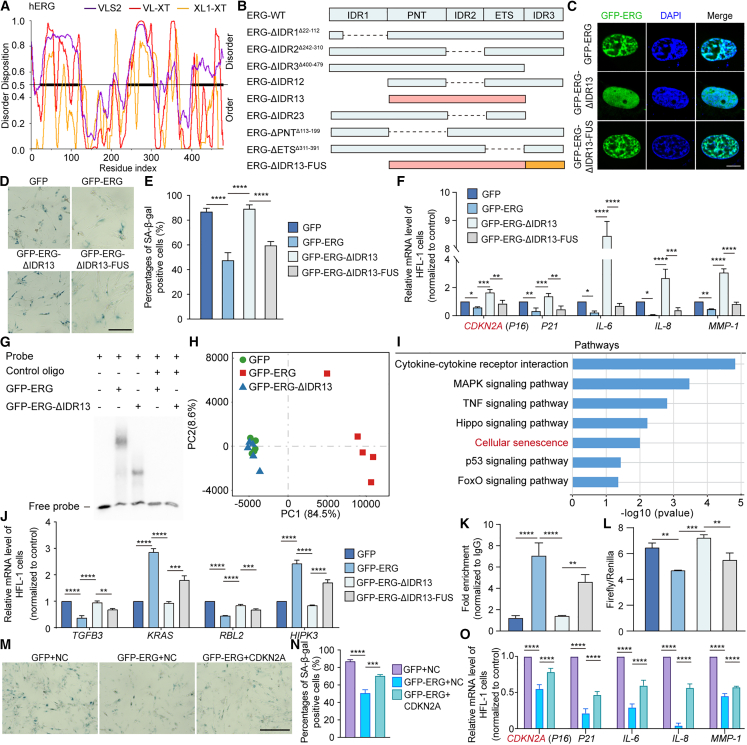
Liquid-liquid phase separation of ERG mediate HFL-1 cell senescence (A) Disorder features of human ERG (hERG) predicted by PONDR. The horizontal line at 0.5 indicates the cutoff for disordered (>0.5) and ordered (<0.5) regions. VLS2, VL-XT, and XL1-XT represent independent disorder predictors. (B) Schematic diagram of ERG truncation mutants. (C) Immunofluorescence images of HFL-1 cells transduced with lentiviruses expressing GFP-ERG, GFP-ERG-ΔIDR13, and GFP-ERG-ΔIDR13-FUS. Scale bars, 10 μm (D) Representative images of SA-β-gal staining of HFL-1 cells transduced with lentiviruses expressing GFP, GFP-ERG, GFP-ERG-ΔIDR13, and GFP-ERG-ΔIDR13-FUS. Scale bars, 20 μm (E) Quantification of the relative percentages of SA-β-gal-positive cells shown in (D) (*n* = 3 biological replicates, with more than 50 cells quantified per replicate). (F) RT-qPCR analysis of CDKN2A (p16), CDKN1A (p21), and SASP gene expression in HFL-1 cells transduced with lentiviruses expressing GFP-ERG, GFP-ERG-ΔIDR13, and GFP-ERG-ΔIDR13-FUS. (G) EMSA analysis of the direct interaction between ERG or ERG-ΔIDR13 protein and the oligonucleotide probe. (H) PCA of transcriptomic profiles of HFL-1 cells overexpressing GFP, GFP-ERG, or GFP-ERG-ΔIDR13. (I) Representative KEGG pathways enriched among genes activated in HFL-1 cells overexpressing GFP-ERG, compared with genes differentially expressed between GFP and GFP-ERG-ΔIDR13 groups. (J) RT-qPCR analysis of the expression levels of overlapping genes from major pathways and ERG target genes. (K) ChIP-qPCR analysis of ERG enrichment at the CDKN2A promoter in HFL-1 cells overexpressing GFP, GFP-ERG, GFP-ERG-ΔIDR13, or GFP-ERG-ΔIDR13-FUS. (L) Luciferase reporter assay of the CDKN2A promoter in HFL-1 cells overexpressing GFP, GFP-ERG, GFP-ERG-ΔIDR13, or GFP-ERG-ΔIDR13-FUS. (M) Representative images of SA-β-gal staining of HFL-1 cells overexpressing GFP-ERG followed by CDKN2A overexpression. Scale bar, 50 μm. (N) Quantification of the relative percentages of SA-β-gal-positive cells shown in (M). (O) RT-qPCR analysis of *CDKN2A* (p16), *CDKN1A* (p21), and SASP gene expression in HFL-1 cells overexpressing GFP-ERG followed by *CDKN2A* overexpression. Data are presented as mean ± SD from three independent biological replicates. Statistical significance was assessed using one-way ANOVA followed by Tukey’s multiple comparisons test. ∗*p* < 0.05; ∗∗*p* < 0.01; ∗∗∗*p* < 0.001; ∗∗∗∗*p* < 0.0001. See also Figure S6.

Intrinsically disordered regions (IDRs) are a common feature of proteins capable of undergoing phase separation.^64^ Sequence analysis identified three IDRs within ERG predicted to drive its LLPS: IDR1 (amino acids 22–112, N terminus), IDR2 (242–310, central region), and IDR3 (400–479, C terminus) (Figure 6A). Structurally, ERG consists of an N-terminal pointed (PNT) domain (113–199) and a C-terminal ETS DNA-binding domain (311–391) (Figure 6B).

To pinpoint which region mediates ERG condensation, we generated a series of deletion mutants lacking individual or combined IDRs or functional domains, including ΔIDR1, ΔIDR2, ΔIDR3, ΔIDR12, ΔIDR13, ΔIDR23, ΔPNT, and ΔETS (Figure 6B). *In vitro* assays showed that the deletion of a single IDR partially impaired droplet formation, whereas removal of two IDRs (ΔIDR12, ΔIDR13, or ΔIDR23) completely abolished condensate formation (Figure S6A). In HEK293T cells, most mutants still formed nuclear puncta similar to wild-type ERG, except for ΔIDR13 (Figures S6B and S6C). In HFL-1 cells, ERG-ΔIDR13 also displayed a diffuse nuclear distribution without visible condensates (Figure 6C), indicating that IDR1 and IDR3 act cooperatively to mediate ERG coacervation. Thus, ERG-ΔIDR13 was designated as the LLPS-deficient variant for subsequent analyses.

Previous studies have shown that the intrinsically disordered N-terminal domain of FUS can drive LLPS and has been used to restore phase separation in other proteins.^65^^,^^66^ Accordingly, fusion of FUS IDR to ERG-ΔIDR13 successfully rescued condensate formation both *in vitro* and in cells (Figures 6B, 6C, and S6A–S6C).

To examine whether ERG-mediated LLPS influences cellular senescence, GFP-ERG constructs (WT, ΔIDR13, and ΔIDR13-FUS) were expressed in HFL-1- and BLM-treated HEK293T cells. SA-β-gal staining, a hallmark of senescence,^67^ revealed that GFP-ERG and GFP-ERG-ΔIDR13-FUS markedly reduced senescence-associated β-galactosidase activity, whereas GFP-ERG-ΔIDR13 had no detectable effect in either cell type (Figures 6D, 6E, S6D, and S6E).

CDKN2A (p16) and CDKN1A (p21), key regulators of the senescence program and upstream drivers of SASP,^68^ were both downregulated by ERG-WT and ERG-ΔIDR13-FUS, but upregulated by ERG-ΔIDR13 in HFL-1- and BLM-treated HEK293T cells (Figures 6F and S6F). Likewise, expression of other canonical SASP components (IL6, IL8, and MMP1)^69^ was markedly suppressed by ERG-WT and ERG-ΔIDR13-FUS, but remained unchanged upon ERG-ΔIDR13 expression, indicating that LLPS is indispensable for ERG to mitigate cellular senescence (Figures 6F and S6F).

As LLPS often influences TF binding and activity,^70^ we next evaluated the DNA-binding capacity of ERG using electrophoretic mobility shift assays (EMSA). The LLPS-deficient ERG-ΔIDR13 exhibited markedly reduced DNA binding compared with ERG-WT (Figure 6G), suggesting that phase separation enhances ERG’s transcriptional function.

To further assess the transcriptional consequences of ERG condensation, we performed RNA-seq in HFL-1 cells expressing GFP, GFP-ERG, or GFP-ERG-ΔIDR13. PCA and hierarchical clustering revealed a distinct transcriptomic profile in GFP-ERG cells compared with GFP and GFP-ERG-ΔIDR13 (Figures 6H and S6G–S6I). KEGG and GSEA indicated that ERG-regulated genes were enriched in aging-related pathways, particularly cellular senescence and cell cycle regulation (Figures 6I, S6J, and S6K).

From our integrated multi-omics analysis, we identified 1,594 potential ERG target genes. Intersecting these with the cellular senescence pathway yielded six likely direct ERG targets—HIPK3, KRAS, FAS, RBL2, TGFB3, and CDKN2A (Figures S6L and S6M). RT-qPCR validation confirmed differential regulation of these genes in GFP-ERG expressing cells (Figures 6F and 6J). Notably, CDKN2A, FAS, and TGFB3, well-characterized aging-associated genes from the Aging Atlas, were significantly affected. chromatin immunoprecipitation assay followed by quantitative PCR (ChIP-qPCR) analysis further demonstrated that GFP-ERG directly bound the *CDKN2A* promoter, while ERG-ΔIDR13 binding was markedly reduced; this was restored by the FUS IDR fusion (Figure 6K). Consistently, luciferase reporter assays confirmed that ERG repressed CDKN2A transcription (Figure 6L). Moreover, overexpression of CDKN2A rescued senescence phenotypes in ERG-overexpressing HFL-1 cells, as shown by increased SA-β-gal staining and elevated SASP gene expression (Figures 6M–6O).

Together, these findings demonstrate that ERG phase separation is crucial for its transcriptional repression of senescence-associated genes, particularly CDKN2A, thereby mitigating cellular aging through LLPS-dependent chromatin engagement and gene regulation.

## Discussion

Our study defines a distinct chromatin accessibility signature in PBMCs of centenarians, characterized by a global increase in chromatin openness across multiple immune subsets. Notably, this increase does not reflect accelerated senescence as aging usually along with increase chromatin accessibility,^71^ but rather suggests a unique chromatin configuration associated with exceptional longevity. In particular, B cells from centenarians display enhanced accessibility at promoter and enhancer regions that typically close with age,^17^ while closing peaks are enriched in quiescent loci that generally open during aging.^17^ These findings highlight that centenarians maintain an atypical epigenetic state, potentially supporting immune resilience and genomic stability in extreme old age.

Despite the well-established link between chromatin remodeling and transcriptional activity,^72^^,^^73^ our data revealed only a weak correlation between accessibility and gene expression changes, underscoring the complexity of transcriptional regulation in longevity. Consistently, DNA methylation clocks and chromatin accessibility appear to capture distinct layers of aging biology.^74^ However, Only 14 of the 2,109 CpG sites from a published methylation clock^75^ overlapped with our 30,733 PBMC ATAC-seq peaks (data not shown), likely reflecting both technical differences (CpG arrays versus regulatory-element-focused ATAC-seq) and biological differences (whole blood versus immune subsets). These observations suggest that additional regulatory layers, including TF activity,^76^ post-transcriptional modulation^77^ and environmental adaptation,^78^ might contribute to the observed expression patterns. Integrating multiple epigenomic and transcriptomic approaches will therefore be essential to disentangle the multifactorial mechanisms of healthy aging.

Sex-specific analyses revealed that female centenarians exhibited more pronounced chromatin remodeling compared to males, with enrichment of ERG motifs in female-biased open regions. This suggests that ERG-mediated transcriptional regulation may be more active in females who achieve extreme longevity. While the smaller sample size of males might partially contribute, the high overall correlation between age-matched male and female PBMC profiles indicates that these differences largely reflect biological, rather than sampling, effects. These findings underscore the importance of investigating sex-dependent molecular mechanisms in aging and longevity.

Motif enrichment and transcriptomic analyses converged on ERG as a putative longevity-associated TF, characterized by coordinated enrichment in opening peaks and expression in centenarians. ERG is a well-established regulator of vascular endothelial identity, where it maintains chromatin accessibility, stabilizes super-enhancer architecture, and preserves endothelial homeostasis.^79^ Moreover, ERG has been shown to undergo age-associated dysfunction in vascular endothelium, leading to reduced chromatin accessibility and maladaptive transcriptional responses to injury.^80^ These findings highlight ERG as a key chromatin remodeler whose integrity is essential for vascular health across the lifespan. The unexpected upregulation of ERG in PBMCs from centenarians therefore raises intriguing questions regarding its cellular origin and functional relevance in the aging immune system. Although ERG is canonically restricted to endothelial cells,^81^ its induction in centenarians may reflect low-level activation across multiple immune lineages, stress-responsive transcriptional reprogramming, or endothelial-like epigenetic states emerging within peripheral immune cells during extreme aging. Dissecting the precise PBMC subsets that contribute to this signal will require single-cell or lineage-resolved profiling, which was not feasible in the present study due to limited sample availability. Ongoing expansion of centenarian cohorts and higher-resolution epigenomic profiling will be crucial for elucidating whether ERG activation in peripheral immune cells represents a compensatory chromatin state, a circulation-linked endothelial signature, or a broader adaptive mechanism supporting healthy longevity. Which PBMC subsets might contribute to this ERG upregulation? However, due to the limited availability of PBMC samples from centenarians, we were unable to perform further subset-specific experiments at present. We are now actively collecting additional samples to address this question in future work. It is plausible that ERG expression in centenarian PBMCs represents low or transient activation across multiple immune populations, possibly reflecting endothelial-like transcriptional reprogramming. Given that ERG is a chromatin organizer in endothelial cells, such reactivation could facilitate adaptive chromatin regulation that supports immune homeostasis and stress resistance in extreme old age. Nevertheless, as aberrant overexpression of ERG due to chromosomal translocations is known to be oncogenic in prostate epithelial cells,^82^ its role in PBMCs must be interpreted with caution; the effects of ERG in the hematopoietic context may be distinct from those in epithelial tissues and merit further investigation.

Functionally, we demonstrated that ERG undergoes LLPS both *in vitro* and *in vivo*, driven primarily by its IDRs (IDR1 and IDR3). This biophysical property was found to be essential for its chromatin regulatory activity, as the LLPS-deficient mutant ERG-ΔIDR13 failed to alleviate cellular senescence and even aggravated senescence-associated phenotypes. These results reveal that ERG’s phase separation underlies its ability to organize chromatin and modulate transcriptional programs relevant to aging, linking protein biophysics to transcriptional control and longevity mechanisms.

In conclusion, our findings uncover ERG as a longevity-associated TF that integrates chromatin remodeling, gene regulation, and LLPS to promote cellular and immune function in extreme aging. The unexpected expression of ERG in PBMCs opens new avenues for understanding adaptive chromatin mechanisms in longevity. Future studies using single-cell and lineage-resolved approaches will be critical to define the cellular origin and functional consequences of ERG activity in the immune system. Moreover, strategies aimed at modulating ERG condensate dynamics may provide new opportunities to promote healthy aging through targeted regulation of chromatin organization.

### Limitations of the study

This study has several limitations that warrant consideration. First, the limited availability of PBMC samples from centenarians constrained certain analyses, including cell subset-specific profiling and protein-level validation of key findings such as ERG expression. Second, bulk ATAC-seq and RNA-seq data provide averaged signals across heterogeneous immune cell populations, which may obscure lineage-specific regulatory changes. Future single-cell multiomic analyses will be essential to dissect the precise cellular contexts and functional consequences of the chromatin and transcriptional features identified here. Third, while our experimental models revealed that ERG phase separation mitigates cellular senescence, these systems may not fully recapitulate the complex *in vivo* environment of centenarians. Further studies using primary human cells and physiological aging models will be needed to validate and extend these findings.

## Resource availability

### Lead contact

Further information and requests for resources and reagents should be directed to the lead contact, Hao Jiang (haojiang@scu.edu.cn).

### Materials availability

This study does not generate new unique reagents.

### Data and code availability

• Bulk ATAC-seq and RNA-seq data of PBMCs of centenarian and control groups generated in this study have been deposited in the Sequence Read Archive (SRA) repository database under accession code BioProject: PRJNA1253085 and BioProject: PRJNA1256436, respectively, and are publicly available. Bulk RNA-seq data of HFL-1 cells overexpressing GFP, GFP-ERG, or GFP-ERG-ΔIDR13 have been deposited at Gene Expression Omnibus (GEO) with accession code GEO: GSE294253 and are publicly available.
• This study did not generate new code. All software used for data analysis is listed in the key resources table.
• Original western blot images have been deposited at Mendeley at Mendeley Data: http://www.doi.org/10.17632/p76df36xns.1 and are publicly available. Any additional information required to reanalyze the data reported in this paper is available from the lead contact upon request.

## Acknowledgments

We would like to thank the Yanjiang District People’s Government of Ziyang City, some staff of the Eighth People’s Hospital of Chengdu City, and the Wenjiang District People’s Government of Chengdu City for their efforts in recruit centenarians. This research was funded by the 10.13039/501100012166National Key Research and Development Program of China (2024YFE0104700); the 10.13039/501100001809National Natural Science Foundation of China (32090043); the National Clinical Research Center for Geriatrics, West China Hospital, Sichuan University (Z20201009, Z2023YY003, Z2023LC005, and Z2024JC003); the 1·3·5 Project for Disciplines of Excellence, West China Hospital, Sichuan University (ZYYC25009); and the 10.13039/501100018542Natural Science Foundation of Sichuan Province, China (2024NSFSC1604).

## Author contributions

Conceptualization, H. Jiang, B.D., L.P., Z.Z., and H.Z.; methods, L.P., Z.Z., H.Z., R.O., R.G., Z.D., X.W., C.X., M.G., L.Z., H. Jin, S.Q., F.H., J.C., H.L., and Y.Z.; data analyses, L.P., Z.Z., and H.Z.; writing – original draft preparation, H. Jiang, L.P., and H.Z.; writing – review and editing, all co-authors; funding acquisition, H. Jiang, B.D., Z.Z., C.X., and L.Z. All authors have read and agreed to the published version of the manuscript.

## Declaration of interests

The authors declare no competing interests.

## STAR★Methods

### Key resources table

**Table:** 

REAGENT or RESOURCE	SOURCE	IDENTIFIER
**Antibodies**		

GFP	Abcam	Cat# ab290; RRID:AB_303395
GFP	Proteintech	Cat# 50430-2-AP; RRID:AB_11042881
ERG	Abcam	Cat# ab92513; RRID:AB_2630401
CDKN2A	Abcam	Cat# ab108349; RRID:AB_10858268
β-actin	ServiceBio	Cat# GB11001; RRID:AB_2801259
lgG	Solarbio	Cat# K1031R-G10; RRID: AB_3720091
lgG	Thermo Fisher Scientific	Cat# A-11008; RRID:AB_143165
lgG	Jackson ImmunoResearch Labs	Cat# 111-035-003; RRID:AB_2313567

**Bacterial and virus strains**		

DH5a	Transgen Biotech	Cat#:CD201
BL21 (DE3)	Transgen Biotech	Cat# CD601

**Biological samples**		

Human blood	This paper	N/A

**Chemicals, peptides, and recombinant proteins**		

Uni Seamless Cloning and Assembly Kit	Transgene	Cat# CU101
Q5® Site-Directed Mutagenesis Kit	New England Biolabs	Cat# E0554S
F12-K	Gibco	Cat# 30-2004
DMEM	Gibco	Cat# C11995500BT
FBS	Excell	Cat# FSP500
Opti-MEM	Thermo Fisher Scientific	Cat# 31985070
Lipo3000	Thermo Fisher Scientific	Cat# L3000015
Ni-NTA Agarose	QIAGEN	Cat# 30230
Ficoll-Histopaque	Sigma-Aldrich	Cat# 10771
SA–β-gal staining kit	Beyotime	Cat# C0602
Bleomycin	Aladdin	Cat# B107423

**Critical commercial assays**		

TruePrep DNA Library Prep Kit V2 for illumia	Vazyme	Cat# TD501

**Deposited data**		

Bulk ATAC-seq data of PBMCs of centenarian and control groups	BioProject	BioProject: PRJNA1253085
Bulk RNA-seq data of PBMCs of centenarian and control groups	BioProject	BioProject: PRJNA1256436
Bulk RNA-seq data of HFL-1 cells overexpressing GFP, GFP-ERG or GFP-ERG-ΔIDR13	Gene Expression Omnibus (GEO)	GEO: GSE294253
Original western blot images	Mendeley	Mendeley DATA: http://www.doi.org/10.17632/p76df36xns.1

**Experimental models: Cell lines**		

HEK293T	ATCC	RRID:CVCL_0063
HFL-1	Pricella	Cat# CL-0106

**Oligonucleotides**		

Primers for protein expression constructs, knockdown, qPCR and ChIP-qPCR	This paper	Table S2

**Software and algorithms**		

Graphpad prism 10.1.2	GraphPad	https://www.graphpad.com/resources
trim galore 0.6.7	Github	https://github.com/FelixKrueger/TrimGalore
Bowtie2 2.4.4	Github	https://github.com/BenLangmead/bowtie2
Sambamba 0.6.6	Github	https://github.com/biod/sambamba
MACS2 2.2.7.1	PYPI	https://pypi.org/project/MACS2/
TOBIAS 0.14.0	Github	https://github.com/loosolab/TOBIAS
HOMER 4.11	Benner Lab	http://homer.ucsd.edu/homer/
R 4.3.0	R Consortim	https://www.r-project.org/
GREAT 4.0.4	Bejerano Lab	http://great.stanford.edu/public/html/
LAS X	Leica	N/A
ImageJ 1.54	ImageJ	https://imagej.nih.gov/ij/
QuantaSoft v1.7	Bio-Rad	https://www.bio-rad.com/

**Other**		

TCS-SP8 confocal microscope	Leica	Leica TCS-SP8
Upright Microscopes	Leica	Leica DM6 B
600 MHz nuclear magnetic resonance spectrometer	Bruker	Bruker AvanceIII 600 MHz
Droplet Digital PCR System	Bio-Rad	Bio-Rad QX200

### Experimental model and study participant details

#### Human blood samples

All experiments using human samples in this study were approved by the Ethics Committee on Biomedical Research, West China Hospital of Sichuan University (No. 2020-781). Informed consent was obtained from all donors. A total of 76 community-dwelling healthy volunteers were recruited, including 44 controls (ages 53–73; 29 women, 15 men) and 32 centenarians (ages 99–106; 21 women, 11 men).

All information regarding gender, age, and ethnicity of the volunteers is available in Table S1.

### Method details

#### Isolation of PBMCs

Fresh whole blood from centenarians and controls was collected in 5-mL tubes containing ethylene diamine tetraacetic acid (EDTA). PBMCs were isolated from whole blood within 8 h of sample collection by Ficoll-Histopaque (10771; Sigma-Aldrich, St. Louis, MO) density-gradient centrifugation. Red blood cells were removed by RBC lysis buffer (eBioscience). Cells were pelleted by centrifugation at 500 rpm for 5 min and resuspended in isolation buffer (0.5% BSA, 2 mM EDTA, 4.5 mg/mL D-glucose in PBS). Cell number and viability were measured using an automated cell counter (Countstar Altair).

#### ATAC-seq library generation and preprocessing

ATAC-seq was performed as previously described. 50,000 unfixed nuclei were tagged using Tn5 transposase (TruePrep DNA Library Prep Kit V2 for Illumina TD501; Vazyme) for 30 min at 37°C, and the resulting library fragments were purified using a Qiagen MinElute kit. Libraries were amplified by 10–12 PCR cycles, purified using a Qiagen PCR cleanup kit, and finally sequenced on an Illumina HiSeq 2500 with a minimum read length of 75 bp to a minimum depth of 25 million reads per sample. Two technical replicates were processed per biological sample.

ATAC-seq sequences were quality filtered using trim galore, and trimmed reads were mapped to the GRCh38 (hg38) human reference sequence using bowtie2. After alignment, technical replicates were merged, and all further analyses were performed on these merged data. For peak calling, MACS2^83^ was used with no-model, 100-bp shift, 200-bp extension, and broad peaks options. Only peaks called with a peak score (q-value) of 1% or better were kept from each sample, and the selected peaks were merged into a consensus peak set using the Bedtools multiinter and merge tools. Only peaks called on autosomal chromosomes were used in this study. We further filtered consensus peaks to avoid likely false positives by only including those peaks overlapping more than 20 short reads in at least one sample and peaks for which the maximum read count did not exceed 500 cpm to account for regions that are potential artifacts. Finally, we excluded peaks overlapping blacklisted regions as defined by the ENCODE mappability criteria developed for DNase assays (https://hgdownload.soe.ucsc.edu/gbdb/hg38/problematic/encBlacklist.bb).

#### RNA-seq library generation and preprocessing

Total RNA was isolated from PBMCs using RNeasy (Qiagen) kits following the manufacturer’s protocols. During RNA isolation, DNase treatment was additionally performed using the RNase-free DNase set (Qiagen). RNA quality was checked using Agilent RNA 6000 Nano kit and Agilent 2100 Expert bioanalyzer (Agilent Technologies). RNA quality was reported as a score from A to D, and samples falling below the threshold of B were omitted from the study. cDNA libraries were prepared using a TruSeq Stranded Total RNA LT Sample Prep kit with Ribo-Zero Gold (Illumina), a Kapa Stranded mRNA-Seq Library Prep kit (Kapa Biosystems), or NuGEN Ovation RNA-seq v2 (NuGEN) according to the manufacturer’s instructions using 100 or 500 ng of total RNA. Final libraries were analyzed on a Bioanalyzer DNA 1000 chip (Agilent Technologies). Paired-end sequencing (2 × 75 bp or 2 × 100 bp) of stranded total RNA libraries was performed in an Illumina HiSeq2500 using SBS v3 sequencing reagents. Quality control of the raw sequencing data was performed using the FASTQC tool, which computes read quality using a summary of per-base quality defined using the probability of an incorrect base call. According to our quality criteria, reads with more than 30% of their nucleotides with a Phred score less than 30 were removed, whereas samples with more than 20% of such low-quality reads were dropped from analyses. None of the samples used in this study were dropped after quality control. Reads from samples that passed the quality criteria were quality trimmed and filtered using trimmomatic. High-quality reads were then used to estimate transcript abundance using RSEM.

#### Differential analysis

The R package DESeq2 was used to identify differentially accessibility chromatin regions from ATAC-seq and differentially expressed genes from RNA-seq data between samples of controls and centenarians. In addition to age group, our models included sex in which the sample was collected as covariates because it was determined using principal variance component analysis that these factors account for a sizable fraction of the variance in read counts.

#### Peak annotation and downstream analyses

We annotated ATAC-seq peaks with regard to functional and positional information by multiple data sources as previous reports. HOMER was used to annotate peaks as “promoter” (i.e., within 2 kb of known TSS), “intergenic,” “intronic,” and other positional categories. For functional annotation of peaks, we used a simplified scheme integrating public chromatin states calculated for major PBMC subpopulations with ChromHMM from Roadmap Epigenomics20, Blueprint Epigenome, and a third reference data. We first intersected the ChromHMM-generated states with our set of consensus peaks and solved conflicting cases where multiple chromatin states overlap the same ATAC-seq peak so that each peak was assigned a single annotation, according to the following priority rules: Active Enhancer > Genic Enhancer > Bivalent TSS > Weak Enhancer > Bivalent Enhancer > PolyComb repressed > TSS Flanking > Transcription > ZNF Genes and repeats > Heterochromatin > Quiescent/Low signal. Then, to facilitate interpretation and visualization, we simplified the original sets of chromatin states to a scheme with 6 pooled meta-states, namely (1) TSS, collecting active, flanking, and bivalent TSS states; (2) Enhancer, pooling active, weak and bivalent enhancer states; (3) Repressed PolyComb, combining both weak and strong PolyComb states; (4) Transcription, including both weak and strong transcription states, (5) the quiescent chromHMM state; and (6) other states (ZNF, heterochromatin) combined together. To annotate peaks as cell-specific for a given subset obtained from one of the three datasets listed above, we determined for each peak whether it was annotated as an active promoter or an active enhancer in a single-cell population or lineage, and in such cases labeled the peak accordingly as cell- or lineage specific.

Further functional enrichment analyses were performed using ClueGO (Bindea et al., 2009) to test for overrepresentation of GO: Immune System Process terms using GO term fusion option and WikiPathways pathways among genes associated to differentially open peaks. In addition to testing for enriched gene sets, ClueGO combines GO terms and pathways into functionally relevant meta-sets based on the rate of shared genes among terms, allowing for an efficient assessment of enriched categories as well as their potential interactions, as inferred from sets of shared genes. We applied these methods separately to peaks significantly closing and opening between age groups to investigate the degree to which these two sets of peaks were associated to unique signatures. We only listed terms that are significant at a *p* value of 0.05 after Bonferroni step-down correction. In addition, we used ClueGO to annotate the afore mentioned immunological coexpression modules that were originally associated with unknown function.

#### Congruence between chromatin accessibility and transcription data

ATAC-seq and RNA-seq data were normalized to protein-coding transcripts and annotated using ENSEMBL GRCh38 gene symbols. To integrate chromatin accessibility with transcriptional output, we constructed a paired ATAC-seq/RNA-seq dataset by assigning promoter peaks to their nearest gene based on transcription start site (TSS) annotations. Promoters were defined as ±1,000 bp flanking each TSS. When a promoter peak overlapped with multiple expressed genes, all corresponding peak–gene pairs were retained for visualization. In addition to promoter-associated regions, we incorporated distal regulatory annotations. Distal peaks were assigned to genes based on HOMER-derived enhancer or intergenic annotations, as well as GREAT^34^ (v4.0.4) annotations using the “single nearest gene” model (proximal: 5 kb upstream and 1 kb downstream; distal: up to 1,000 kb). These peak–gene associations were subsequently integrated with RNA-seq expression profiles for joint analysis.

#### TF motif enrichment and footprinting analysis

To examine the enrichment of known TF binding motifs in DARs, BEDTools v2.28.0 was used to obtain the ATAC-seq peak summits located in DA regions, and then “findMotifsGenome.pl” in HOMER was employed to find known motifs and TF binding sites surrounding peak summits with the option-size −200,200. HOMER was also used to identify potential target DEGs and to calculate the occurrence probability of a certain TF within a 1 kb region flanking peak summits (from −500 to +500 bp). After motif enrichment analysis, the findMotifsGenome.pl script was used to find the binding sites of a certain TF according to the tutorial instructions. We defined the potential target DE genes of a certain TF when their corresponding peaks were annotated as promoters. Besides, footprinting analyses were performed using TOBIAS.

#### Functional annotation

We used GO terms to characterize genes annotated to DA peaks and DE genes. After finding numerous GO annotations for the same gene, we prioritized terms based on the following order: immunity > metabolic > transcription, translation > migration > mitochondria > axon > development.

Furthermore, following the process of solving for multiple GO annotations for a given gene, we prioritized terms based on the following order in order to summarize the representation of GO terms among gene annotations for all peaks.

GO, KEGG and GSEA analysis was performed using clusterProfiler (version 4.2.2).

#### Cell culture

The HLF-1 and HEK293T cells (Procell Life Science & Technology) were cultured in F-12 and DMEM (Thermo Fisher Scientific) supplemented with 10% (vol/vol) FBS (Excel), respectively. Cells were grown at 37°C in a 5% CO2 humidified chamber.

#### Identification of potential TF target genes

Potential ERG or NFE2 target genes were identified through a motif-based approach using HOMER. First, ERG or NFE2 binding motifs enriched in differentially accessible (DA) peaks were obtained from the HOMER motif discovery output. The genomic locations of these ERG or NFE2 motifs were then extracted from the HOMER annotation files, and the peaks containing ERG or NFE2 motifs were assigned to their nearest genes using annotatePeaks.pl. To further characterize these candidates, we integrated RNA-seq data and compared the expression levels of the assigned genes between centenarians and age-matched controls. Differential expression analysis was used to determine whether these genes showed significant transcriptional changes.

#### Immunofluorescence

Cells grown on glass coverslips (Thermo Fisher Scientific) were washed twice with phosphate-buffered saline (PBS) and fixed with 4% paraformaldehyde (PFA) for 20 min at room temperature. After fixation, cells were permeabilized with 0.2% Triton X-100 (Biofrom) in PBS for 30 min. Cells were then blocked with 5% bovine serum albumin (BSA) in PBS for 1 h at room temperature. Following blocking, cells were incubated with primary antibody against ERG (Cat#ab92513, 1:50 dilution; Abcam) in blocking buffer at 4°C overnight. The next day, cells were washed three times with PBS containing 0.1% Tween 20 (PBST), 10 min each wash. Cells were then incubated with fluorochrome-conjugated secondary antibody (Goat anti-Rabbit IgG (H + L) Highly Cross-Adsorbed, Alexa Fluor 488; 1:1000 dilution; Cat#A-11008; Thermo Fisher Scientific) in blocking buffer for 1 h at room temperature, protected from light. After secondary antibody incubation, cells were washed again three times with PBST, 10 min each. Nuclei were counterstained with DAPI solution (1 μg/mL) for 5 min, followed by a final wash with PBS. Finally, coverslips were mounted onto glass slides using ProLong Gold Antifade Mountant (Thermo Fisher Scientific) and sealed. Images were acquired using either a Leica fluorescence stereo microscope or a Leica TCS-SP8 confocal microscope.

#### 1,6-Hexanediol (1,6-HD) assay of phase separation

For phase separation *in vivo*, 1,6-hexanediol (sigma) was added to the medium until the concentration of 1,6-HD was 5%. Cells seeded on coverslips were incubated for 7 min in the medium containing 1,6-HD, as the 1,6-HD incubation group for immunofluorescence. Cells seeded on coverslips were incubated in the medium containing 1,6-HD for 7 min and then incubated in the medium without 1,6-HD, as the wash out group for immunofluorescence.

#### Plasmids constructs

All plasmids mentioned were constructed through homologous recombination by using Uni Seamless Cloning and Assembly Kit (Transgene, CU101). To express 6×His-tagged fusion protein in bacteria, the cDNA encoding ERG was cloned into pET-20b at XhoI site. To create His-GFP-ERG, we first inserted GFP into pET32a vector at SalI site, further, the cDNA encodes ERG was cloned into pET32a -GFP at SalI site. The ΔIDR1,ΔIDR2,ΔIDR3, ΔIDR12, ΔIDR13,ΔIDR23, ΔPNT, and ΔETS were created by using Q5 Site-Directed Mutagenesis Kit (NEB, E0554S) as manual instruction. To create GFP-tagged ERG wildtype or truncations expression in cells, the corresponding cDNAs were sub-cloned into pLVx vector at BamHI site. The DNA fragment of ERG-ΔIDR13-FUS was synthesized by Sangon Biotech Co., Ltd (Shanghai, China) and cloned into pET-20b at XhoI site or pLVx vector at BamHI site. The DNA fragment of promoter region of *CDKN2A* was synthesized by Sangon Biotech Co., Ltd (Shanghai, China) and cloned into pGL3-basic vector at HindIII site. Primer sequences are provided in Table S2.

#### Lentivirus packaging and viral transduction

293T cells (80% full) was cultured in a 10 cm Petri dish. pLVX-AcGFP1-C1 vector were used to stably express ERG and the mutants in HFL-1 cells. The plasmids and helper plasmids pCMV-dR8.91, pMD2.G were transfected into 293T cells by lipo8000 (Beyotime). The fluid was changed at 8 h and the virus supernatant was collected at 48 h respectively. The virus was concentrated with polyethylene glycol (PEG) and collected by centrifugation 24 h later for virus titer detection. Lentivirus infected HFL-1 cells was added to the medium containing 1:1000 polyBrene (Solarbio). The efficiency of virus infection was observed under fluorescence microscope, and the cell infection rate reached more than 90%.

#### Protein purification

For expression of His-ERG, His- AcGFP-ERG, His-ERG-ΔIDR2, His-ERG-ΔIDR3, His-ERG-ΔIDR12, His-ERG-ΔIDR13 and His-ERG-ΔIDR23, plasmids were transformed into BL21 (DE3). A single colony was selected and cultured in 10 mL LB medium. After 16 h, the colony was poured into 1L fresh LB medium and cultured at 37°C and 220 rpm. When OD600 reached 0.6–0.8, 1 mM IPTG was added to induce protein expression. Incubated at 16°C, 220 rpm for 16 h, centrifuged at 4°C, 3800 rpm to collect bacteria. Lysis buffer (50 mM NaH2PO4, 300 mM NaCl, 10 mM imidazole, 1 mM PMSF, Ph 8) was added to the bacteria and sonicate for 15 min, and the supernatant was received at 4°C, 12000 rpm. The supernatants are added to the columns with the Ni-NTA beads (Qiagen). After incubated at 4°C for 2 h with rotation, wash the unbound proteins with wash buffer (50 mM NaH2PO4, 300 mM NaCl, 20 mM imidazole, pH 8). Finally, elution buffer (50 mM NaH2PO4, 300 mM NaCl, 500 mM imidazole, pH 8) was used to eluate the protein. The protein collected in elution was concentrated by Amicon Ultra 30K device (Millipore). At the same time, frozen fresh PBS concentration was added and repeated three times to a volume of 200-500ul. The purified protein was quantified using an ND-2000C NanoDrop spectrophotometer (NanoDrop Technologies).

#### Droplets assay and FRAP

10 μL of His-ERG wild-type or truncation protein at the indicated concentrations were prepared by diluting the stock solutions into prechilled PBS. PEG 6000 was added last to a final concentration of 10% (v/v). The mixture was loading into a flow cell made with two pieces of double sticky scotch taps and the whole device was heated to 37°C for 2 min followed by imaging immediately by using differential interference contrast (DIC) microscope (Leica DM6 B). FRAP assay in cells were performed on inverted confocal microscope (Leica SP8) with 63×oil objective. For His-AcGFP-ERG FRAP, the assay was performed on inverted confocal microscope (Leica SP8) with 63× oil objective. Recovery was recorded for the indicated time.

#### Transmission electron microscopy (TEM)

Samples were fixed with 2% paraformaldehyde and 2% glutaraldehyde in 0.1 M phosphate buffer (pH 7.4) at 37 °C for 30 min, and then at 4 °C for 30 min. Afterward, they were fixed with 2% glutaraldehyde 0.1 M phosphate buffer (pH 7.4) at 4 °C overnight. The cells were then washed with the same buffer and post-fixed with 1% osmium tetroxide (OsO4) in the same buffer for 1 h. The samples were then dehydrated for 1 h in a graded ethanol solution and embedded in a resin (Epon-812) for 2 days and polymerized at 60 °C for 48 h. Ultrathin sections (70-nm thickness) were made with a diamond knife on a Leica Ultracut UCT ultramicrotome (Leica), and then mounted on copper grids. Sections were stained with 2% uranyl acetate for 15 min and lead stain solution for 2 min (Merck). Images were acquired with the JEM-1400FLASH electron microscope (JEOL).

#### Immunoelectron microscopy

The immunoelectron microscopy was performed according to a pre-embedding immunogold staining protocol.^84^ HEK293 cells were grown on coverslips, extracted for 5 min in extraction buffer (PEM buffer, 10 μm taxol, 0.1% Triton X-100) and fixed with 2% PFA and 0.1% GA in PBS. Cells were incubated with anti-GFP antibody (Proteintech, Cat#50430-2-AP) and Nanogold-conjugated anti-IgG antibody (Solarbio, Cat#: K1031R-G10) overnight at 4 °C. For observation, the HEK293 cells were transfected with pLVx-GFP-ERG. The cells were processed according to a standard EM sample preparation procedure as in TEM part, and dehydrated and embedded in Epon resin. The samples were observed with a TEM (JEOL).

#### RNA interference

For gene knockdown, cells were seeded in 6-well plates at a density of 2×10^5^ cells per well. The next day, cells at approximately 70% confluence were transfected with 50 nM of target-specific siRNA or a negative control siRNA (Shanghai, GenePharma) using Lipofectamine RNAiMAX Transfection Reagent (Thermo Fisher Scientific) according to the manufacturer’s protocol. Specifically, siRNA and RNAiMAX were diluted separately in Opti-MEM medium, mixed, incubated for 20 min at room temperature to form complexes, and then added dropwise to the culture medium. Cells were harvested for RNA or protein analysis 72 h post-transfection to assess knockdown efficiency. The siRNA sequences targeting ERG are listed in Table S2.

#### Western blot

Cells from each group were lysed on ice using RIPA lysis buffer (P0013B, Beyotime) supplemented with 1 mM PMSF (Servicebio) for 30 min. Lysates were centrifuged at 12,000 × g for 15 min at 4°C, and the supernatants were collected. Protein concentrations were determined using a BCA Protein Assay Kit (P0012, Beyotime). Equal amounts of protein (30 μg per lane) were mixed with 5× SDS-PAGE loading buffer, boiled for 10 min, and separated by electrophoresis on a 12% SDS-PAGE gel at 150 V. Proteins were then transferred onto PVDF membranes (Millipore) in Tris-glycine transfer buffer containing 20% methanol using a wet transfer system at 100 V for 90 min. After transfer, membranes were washed three times (10 min each) with TBST and blocked with 5% non-fat dry milk (Cell Signaling Technology) in TBST for 2 h at room temperature. Membranes were incubated overnight at 4°C with the following primary antibodies diluted in the blocking buffer: anti-ERG (Cat# ab92513, 1:1,000, Abcam), anti-CDKN2A (Cat# ab108349, 1:2,000, abcam), anti-GFP (Cat#ab290, 1:2,000, abcam), and anti-β-actin (Cat# GB11001, 1:2,000, ServiceBio) as a loading control. After three 10 min washes with TBST, membranes were incubated with Peroxidase-AffiniPure Goat Anti-Rabbit IgG (H + L) (Cat# 111-035-003, 1:10,000, Jackson ImmunoResearch Labs) for 1 h at room temperature. Membranes were washed again three times with TBST. Protein bands were visualized using Ultra Signal Enhanced Chemiluminescent (ECL) Reagent kit (4A Biotech Co., Ltd) and imaged with a Bio-Rad ChemiDoc MP Imaging System.

#### SA-β-gal staining

The SA-β-Gal staining of HFL-1 and HEK293T cells was done using an SA–β-gal staining kit (Beyotime, C0602). In brief, HFL-1 and HEK293T cells were washed with PBS before fixation with a buffer containing 2% (w/v) formaldehyde and 0.2% (w/v) glutaraldehyde for 5 min. After washing with PBS for twice, the cells were incubated at 37 °C overnight with staining buffer containing 1 mg/mL X-gal. The optical microscope was used to obtain SA-β-Gal-stained cells, and the percentage of SA-β-Gal-positive cells was calculated.

#### Electrophoretic mobility shift assay (EMSA)

EMSA was performed using Chemiluminescent EMSA Kit (Beyotime). Briefly, 0.1 μM each protein incubated with 0.1 μM biotin-labeled probe in binding buffer for 30 min at room temperature. Meanwhile, reactions contained 0.1 μM excess of the same unlabeled probe were used to determine specific binding. Then the reaction mixtures were separated in a 3% nondenaturing polyacrylamide gel in 0.5 × TBE at 80 V for 1 h. Then the DNA/protein complex was transferred to nylon membrane, conjugated with Streptavidin-HRP, visualized with ECL, and detected by the Bio-Rad ChemiDoc Imaging System.

#### Reverse-transcription quantitative PCR (RT-qPCR)

We used Magzol reagent (Magen) according to the included instructions to extract total RNA from cultured cells at 80% confluence. Then, the total RNA was quantified using an ND-2000C NanoDrop spectrophotometer (NanoDrop Technologies). The first-strand cDNA was synthesized from 1 μg of total RNA with HiScript III RT SuperMix for qPCR (vazyme). RT-qPCR was performed with ChamQ Universal SYBR qPCR Master Mix (vazyme) on a C1000 Touch Thermal Cycler (Bio-Rad). Select β-Actin as the reference gene. 2-ΔΔCT method was used for data calculation.

#### Digital droplet PCR (ddPCR)

The ddPCR was conducted on a Bio-Rad QX200 Droplet Digital PCR System following the manufacturer’s protocol. Each 20 μL reaction mixture contained 10 μL ddPCR EvaGreen SuperMix, 2 μL target gene primers (10 μM), 8 μL nuclease-free water, and 1 μL cDNA template. Reaction mixtures were loaded into a droplet generator cartridge, and 70 μL droplet generation oil was added to the adjacent wells for droplet formation using the Bio-Rad droplet generator. Generated droplets (∼40 μL) were transferred into a 96-well PCR plate, sealed with pierceable foil using the PX1 PCR Plate Sealer, and amplified in a T100 Thermal Cycler (Bio-Rad) under the following conditions: 95 °C for 10 min, then 40 cycles of 94 °C for 30 s and 60 °C for 1 min, followed by a final step at 98 °C for 10 min. After amplification, droplets were analyzed using the Bio-Rad Droplet Reader. Data acquisition and quantification were performed with QuantaSoft v1.7 software (Bio-Rad), which determined the fraction of positive droplets and calculated target copy numbers per droplet using a Poisson distribution model with 95% confidence intervals. Thresholds for positive signal detection were manually set based on no-template control (NTC) wells containing nuclease-free water. Primer sequences are provided in the STAR Methods section.

#### ChIP-qPCR

1×10^7^ HFL-1 cells were harvest for the ChIP assay. ChIP was performed using the SimpleChIP Enzymatic Chromatin IP kit (Cell Signaling Technology, #9003S) according to the manufacturer’s directions. The protein and DNA were cross-linked and added into a 15 cm Petri dish containing 20 mL medium with 540 μL of 37% formaldehyde until its final concentration was 1%, and incubated at room temperature for 10 min (cell density for suspended cells should be less than 0.5×10^6^/mL). The crosslinking reaction terminated by adding glycine (10×). Pre-cooled PBS was added to the Petri dish (1% cooktail was added), the cells were scraped off the cells, and nuclear preparation and chromatin digestion continued. The cells were suspended with 1 mL 1× BufferA (250 μL 4× BufferA#7006 + 750 μL ddH2O) and incubated on ice for 10 min. The cell suspension was centrifuged at 2,000g, 4 °C for 5 min, and the supernatant was removed and discarded. 1.1 mL 1× Buffer B (275 μL 4× Buffer B #7007 + 825 μL ddH2O) was prepared, and 1 mL of Buffer B was taken and suspended again. After centrifugation, the supernatant was discarded and repeated concentrated. After centrifugation, supernatant was removed and re-suspended with 100 μL Buffer B. The samples were transferred a 1.5 mL centrifuge tube, and each sample was added with 0.5 μL Micrococcal Nuclease to digest the DNA fragments on ice for 20 min 10 μL 0.5 M EDTA was added to stop digestion, 500 mL lysate per 1.5 mL microcentrifuge tube ultrasound, and the cell membrane was broken with several pulses. Centrifuge at 9,400 g and 4 °C for 5 min to clarify the dissolved matter. Chromatin immunoprecipitation was done by adding 1 × ChIP Buffer 400 μL (40 μL 10 × ChIP Buffer #7008 + 360 μL ddH2O). The sample was incubated at 4 °C overnight with antibodies against GFP (1:50, abcam, Cat # ab390) or Normal Rabbit IgG (1:50, Cat #2729). Complexes were precipitated with ChIP-Grade Protein G Magnetic Beads (Cat #9006). Beads were then washed sequentially with low-salt immune complex wash, high-salt immune complex wash (DNA wash buffer plus with NaCl). Immunoprecipitated chromatin was eluted in ChIP Elution Buffer, incubated at 65 °C for 30 min and then treated with proteinase K and 5 M NaCl for 6 h. At last, DNA was purified by Purification of DNA by centrifugation column as protocol. ChIP (enriched) and control (negative control) DNA samples were amplified by SimpleChIP Universal qPCR Master Mix (Cat #88989).

#### Luciferase reporter assay

Firefly luciferase activity was quantified using the Dual Luciferase Reporter Assay Kit (Vazyme, China, DL101-01) following the manufacturer’s instructions. A total of 300,000 HFL-1 cells were sorted and immediately frozen in liquid nitrogen. Luciferase signals were subsequently detected according to the kit protocol. Simultaneously, 20 μL of the lysate supernatant was collected for protein concentration measurement using a BCA assay. Luciferase activity was normalized to protein concentration to ensure accuracy. Each genotype and condition were analyzed in triplicate.

#### Sequence analysis for protein disorder

We used the PONDR program (http://www.pondr.com/) to analyze disordered regions of ERG. Three predictors (VL-XT, XL1-XT, and VSL2) indicated that ERG possesses intrinsic disorder regions.

### Quantification and statistical analysis

GraphPad Prism 10.1.2 software (GraphPad Software) was used for statistical data analysis. Pairwise comparisons were made using a two-sided Mann–Whitney test. Multiple comparisons were performed using an ordinary one-way analysis of variance (ANOVA) followed by Tukey’s multiple comparisons test. Data are presented as the mean ± standard deviation (SD), unless otherwise indicated. Recovery curves of FRAP assays represent the mean ± standard error of the mean (SEM) from the indicated number of cells or granules, as specified in the figure legends. A *p* value of less than 0.05 was considered significant. ns, not significant; ∗*p* < 0.05, ∗∗*p* < 0.01, ∗∗∗*p* < 0.001, and ∗∗∗∗*p* < 0.0001.
