# Supplementary material for: ERG phase separation attenuates cellular senescence
Source: iScience. 2026 Jan 13;29(2):114678. doi: 10.1016/j.isci.2026.114678 (PMC12907116; doi:10.1016/j.isci.2026.114678)
Supplement: Document S1. Figures S1–S6 and Tables S1 and S2 [file mmc1.pdf]

iScience, Volume *29*

Supplemental information

ERG phase separation attenuates cellular senescence

Lu Pu, Zhiliang Zuo, Hui Zheng, Rui Ou, Ru Gao, Zhaomin Deng, Xiaochu Wu, Chun Xiao, Meiling Ge, Lixing Zhou, Haoran Jin, Shaochong Qi, Fengjuan Hu, Jieli Chen, Hang Li, Yan Zhao, Birong Dong, and Hao Jiang

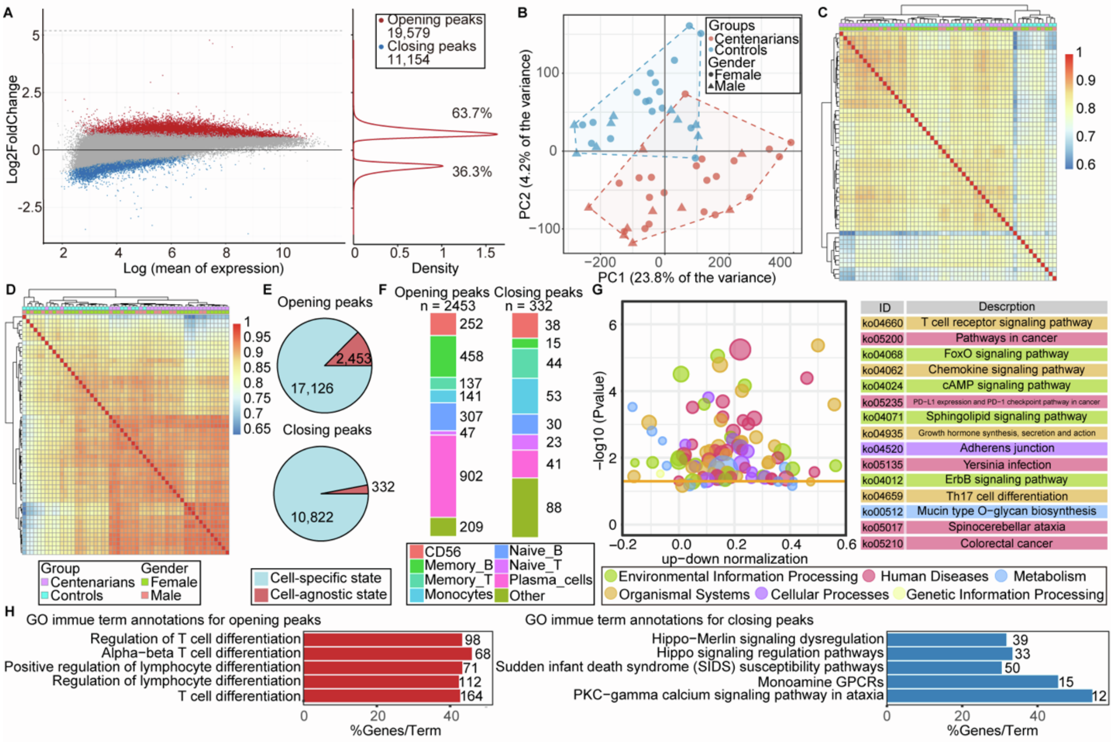

Figure S1. An epigenomic signature of centenarians in PBMCs, related to Figure 1

(A) Plot representing log2 fold change (centenarians-controls) versus mean read count for ATAC-seq peaks. Changes in chromatin accessibility in centenarians. The percentage of open peaks is shown under the density curve in the right panel.

(B) PCA analysis based on all accessible peaks.

(C and D) Heat map showing chromatin profiles for all (C) and DA peaks (D) across PBMC samples of centenarians and controls.

(E) Distribution of cell-specific and cell-agnostic loci among DA peaks based on chromHMM states in PBMCs and immune cell subsets.

(F) Number of cell-specific loci among opening and closing peaks in centenarians.

(G) KEGG pathways of genes associated with DA peaks.

(H) ClueGO GO term enrichment results for opening and closing peaks.

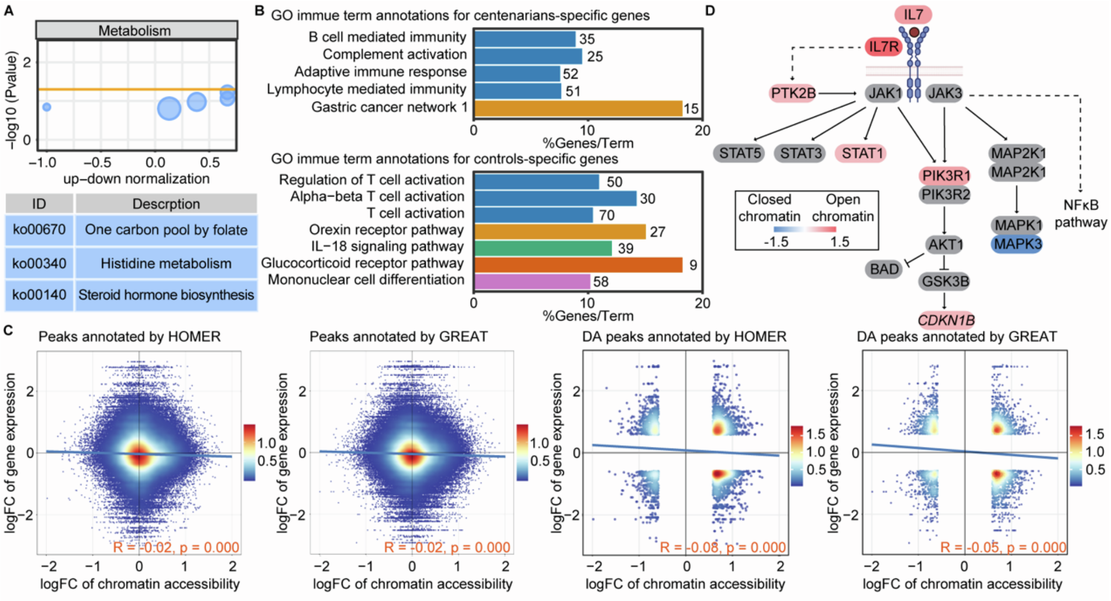

Figure S2. Integrated ATAC-seq and RNA-seq comparison and extended correlation analyses, related to Figure 2

(A) KEGG metabolic pathways of genes associated with DE genes.

(B) ClueGO GO term enrichment results for up- and down- regulated genes.

(C) Correlation of ATAC-seq and RNA-seq. The peaks were annotated by HOMER or GREAT.

(D) Chromatin accessibility of peaks annotated to genes in the IL7 signaling pathway.

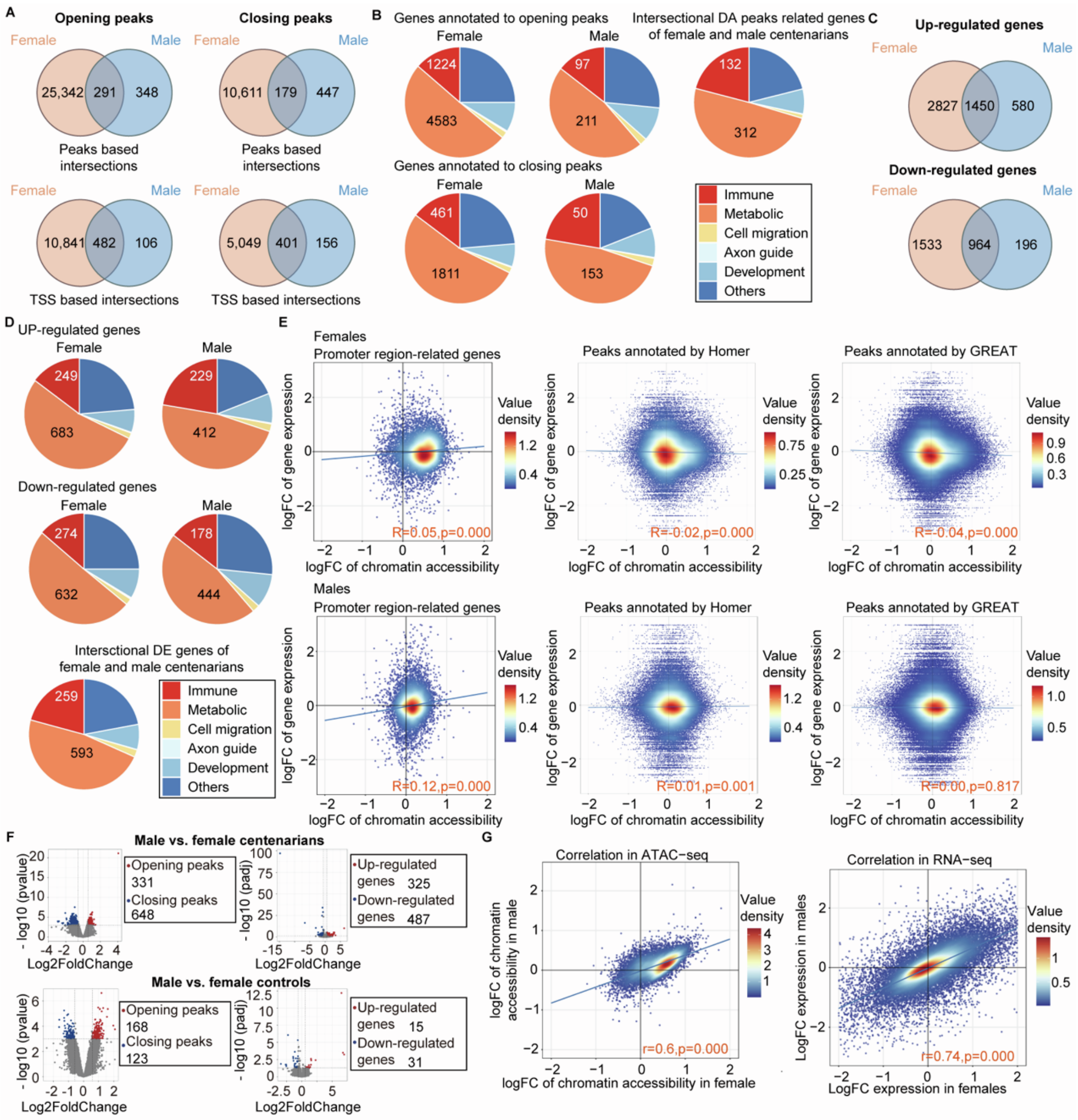

Figure S3. Expanded sex-specific chromatin and transcriptional analyses, related to Figure 3

(A) Overlap between DA peaks (top) and genes associated to DA peaks (bottom) in female/male centenarians compared to controls.

(B) Major GO category annotations of genes associated with opening and closing peaks in female/male centenarians compared to controls and their overlap.

(C) Overlap between DE genes in female/male centenarians compared to controls.

(D) Major GO category annotations of genes associated with up- and down- regulated genes in female/male centenarians compared to controls and their overlap.

(E) Correlation of ATAC-seq and RNA-seq of female/male centenarians. The peaks were annotated by HOMER or GREAT. And genes with chromatin remodeling at gene promoters were annotated by HOMER.

(F) Volcano plots of DA peaks or DE genes between female and male centenarians/controls.

(G) Correlation of ATAC-seq and RNA-seq data between female and male centenarians.

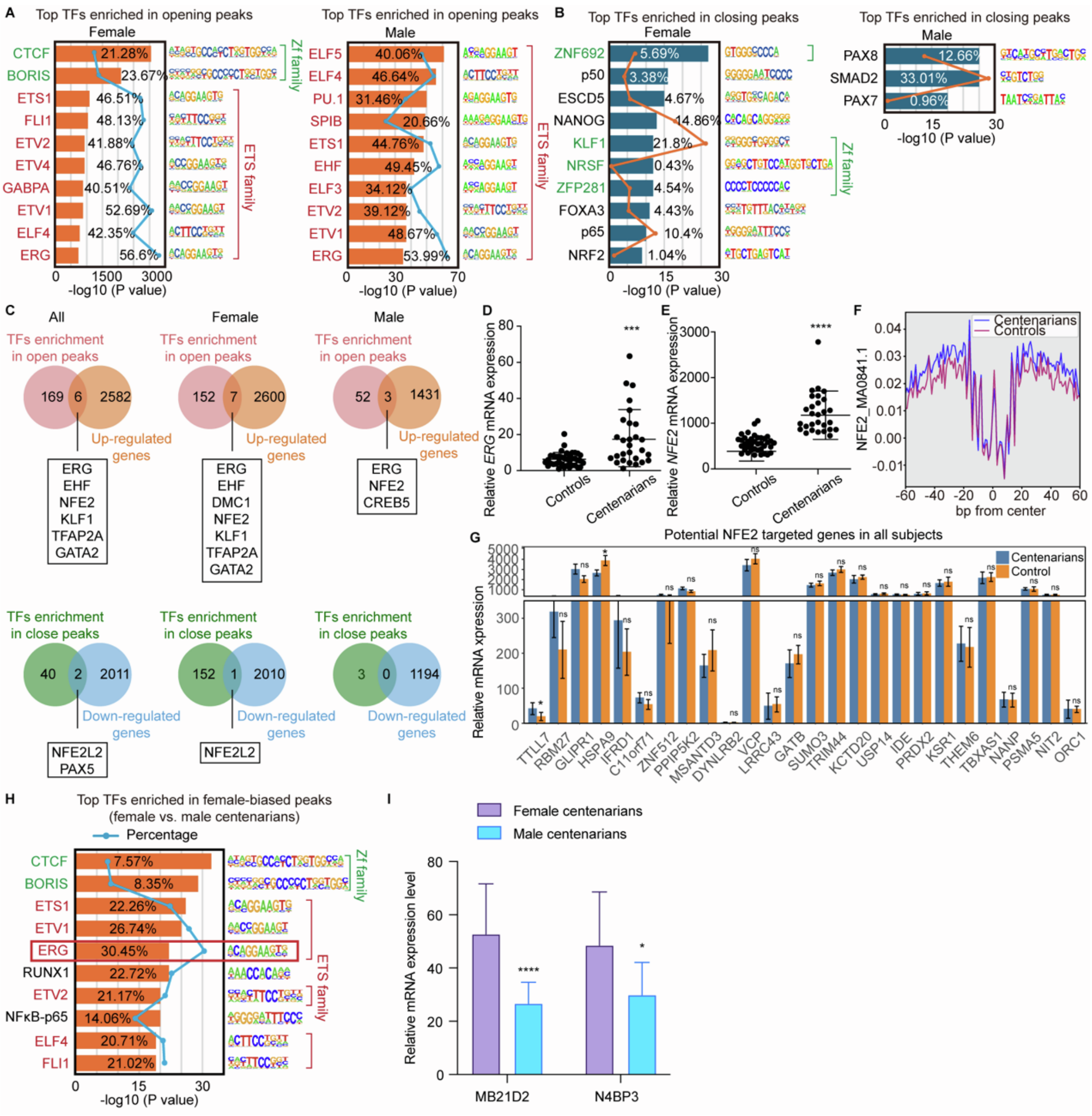

Figure S4. Expanded analyses of ERG motif enrichment in female and male centenarians, related to Figure 4

(A and B) Top enriched known TF motifs of opening (A) and closing (B) peaks in female/male centenarians when compared to controls.

(C) Venn diagram showing overlap of the corresponding TFs for each motif enriched in opening/closing peaks and up-/down- regulated genes in all, female and male centenarians, respectively.

(D and E) Relative *ERG* (E) and *NFE2* (F) mRNA expression from RNA-seq data.

(F) NFE2 motif footprint.

(G) Bar charts showing the expression levels of potential NFE2 target DE genes in DA peaks. Error bars indicate SD.

(H) Top enriched known TF motifs of opening peaks in female centenarians compared to male centenarians.

(I) The expression level of DE genes in female compared to male centenarians potentially regulated by ERG.

Data are presented as mean ± SD. Statistical significance between groups was assessed using

a two-sided Mann–Whitney test. ns, not significant; *p < 0.05; **p < 0.01; ***p < 0.001; ****p < 0.0001.

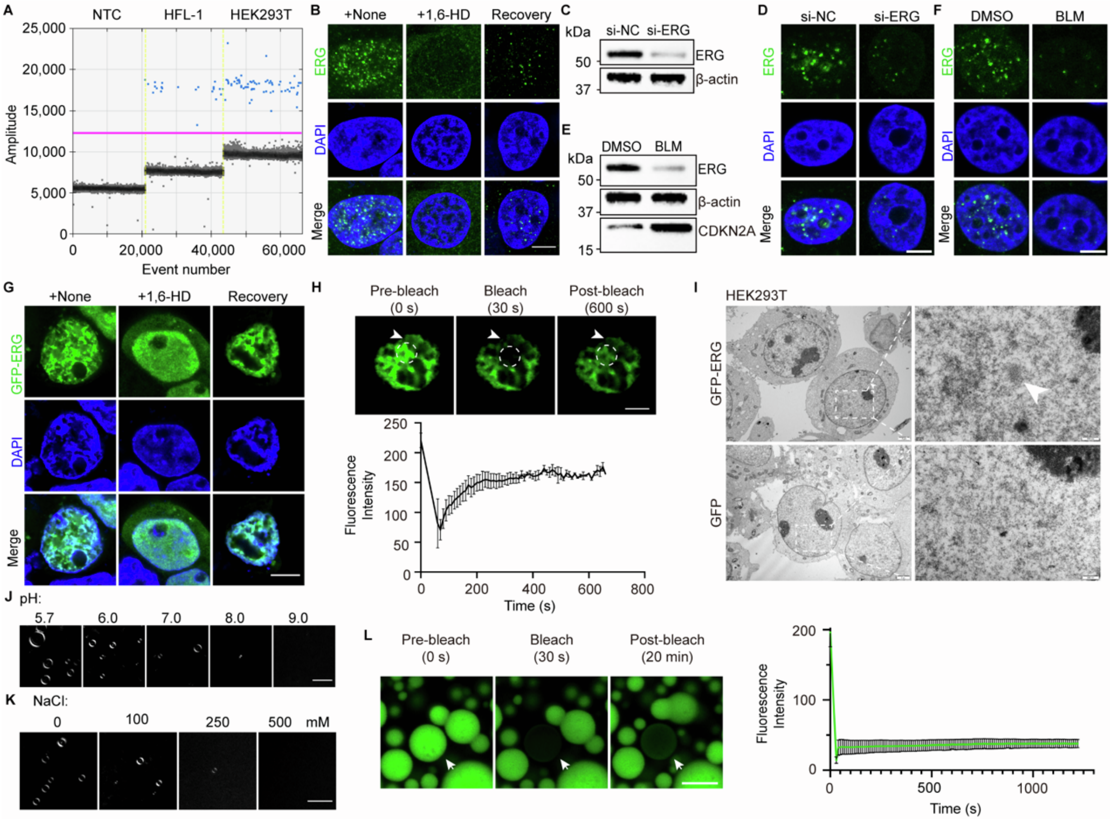

Figure S5. ERG underwent liquid-liquid phase separation in HEK293T cells and in *vitro*, related to Figure 5

(A) Digital droplet PCR analysis of ERG in HFL-1 and HEK293T cells. NTC, No Template Control.

(B) Representative images of endogenous ERG puncta in HEK293T cells. The incubated and wash-out of 1,6-hexanediol (1,6-HD) destroy and re-formation of the condensation in the nuclei. Scale bar, 10 μm.

(C) Immunoblotting analyses of ERG protein expression in HEK293T cells treated with control or ERG RNAi. β-actin, the loading control.

(D) Representative images of endogenous ERG puncta in HEK293T cells treated with control or ERG RNAi. Scale bar, 10 μm.

(E) Immunoblotting analyses of ERG and CDKN2A protein expression in HEK293T cells treated with DMSO or BLM. β-actin, the loading control.

(F) Representative images of endogenous ERG puncta in HEK293T cells treated with DMSO or BLM. Scale bar, 10 μm.

(G) Representative images of GFP-ERG condensation in HEK293T cells. The incubated and wash-out of 1,6-hexanediol (1,6-HD) destroy and re-formation of the condensation in the nuclei. Scale bar, 10 μm.

(H) Fluorescence recovery of GFP-ERG condensation in HEK293T cells after photobleaching. The photobleaching area is indicated by the white circle and arrow (upper panel). Quantification of fluorescence recovery over time from 15 individual cells is shown (lower panel). Data are presented as mean ± SEM. Scale bar, 10 μm.

(I) TEM image of 293T cells overexpressed GFP-ERG. The condensates formed by GFP-ERG are indicated by the white box and arrow. Scale bar, 2 μm (left); 500 nm (right).

(J and K) Formation of ERG droplets underdifferent NaCl concentrations (E) and pH values (F). Scale bar, 10 μm.

(L) Fluorescence recovery of GFP-ERG protein after photobleaching are shown. The photobleaching area is indicated by the white arrow (left panel). Fluorescence recovery curves after photobleaching for GFP-ERG condensates are shown (right panel). Data are presented as mean ± SEM, n = 5 granules. Scale bar, 10 μm.

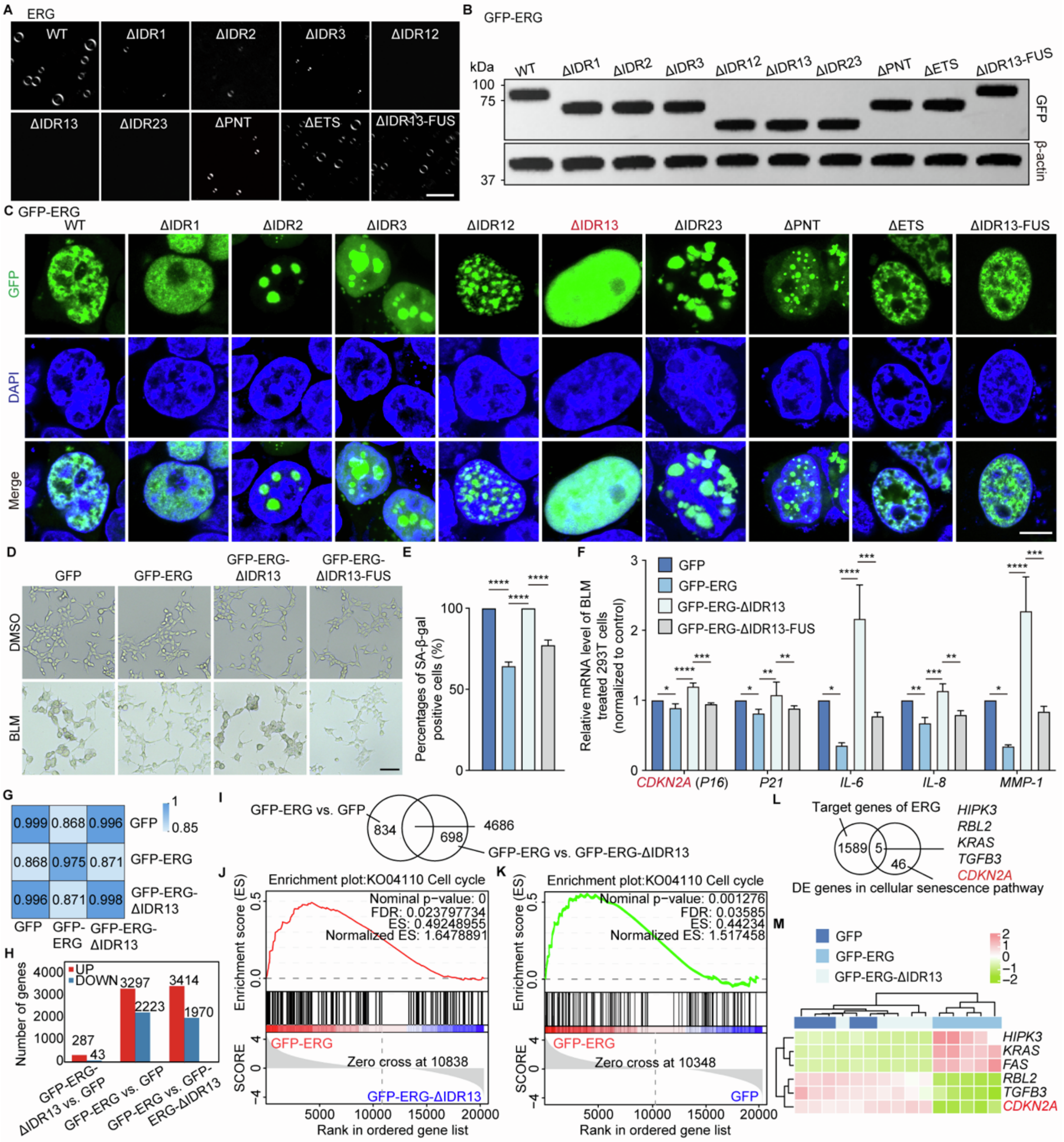

Figure S6. Characterization of ERG LLPS and its function in cellular senescence, related to Figure 6

(A) Droplet formation of protein of ERG truncation mutants. Scale bar, 10 μm.

(B) Immunoblotting analyses of GFP-ERG and its truncation mutants in HEK293T cells. β-actin, the loading control.

(C) Immunofluorescence images of GFP-ERG and its truncation mutants in HEK293T cells. Scale bar, 10 μm.

(D) Representative images of SA-β-Gal staining of HEK293T cells transduced with lentiviruses expressing either GFP, GFP-ERG or GFP-ERG-ΔIDR13 treated with bleomycin (BLM). Scale bar, 50 μm.

(E) Quantitation of the relative percentages of SA-β-Gal-positive cells. n = 3 biological replicates. Over 50 cells were quantified in each replicate.

(F) RT-qPCR for the expression levels of *CDKN2A* (*p16*), *CDKN1A* (*p21*) and SASP genes in HEK293T cells transduced with lentiviruses expressing either GFP, GFP-ERG or GFP-ERG-ΔIDR13 treated with BLM.

(G) Euclidean distance heatmap showing sample repeatability of RNA-seq data in HFL-1 cells expressing either GFP, GFP-ERG or GFP-ERG-ΔIDR13.

(H) Differentially expressed genes among HFL-1 cells expressing GFP, GFP-ERG or GFP-ERG-ΔIDR13. (h) Venn diagram showing overlap of DE genes between cells expressing GFP/GFP-ERG-ΔIDR13 and GFP-ERG.

(I) Overlap of DE genes between GFP-ERG vs GFP and GFP-ERG vs GFP-ERG-ΔIDR13.

(J and K) Enrichment plots from gene set enrichment analysis (GSEA). GSEA was performed to assess enrichment of cell cycle in transcriptomes of cells expressing GFP-ERG vs GFP (J) or GFP-ERG vs GFP-ERG-ΔIDR13 (K).

(L) Overlap of target genes of ERG and DE genes in cellular senescence pathway.

(M) Heatmap showing the relative expression of indicated genes in Figure S6L.

Data are presented as mean ± SD from three independent biological replicates. Statistical significance was assessed using one-way ANOVA followed by Tukey's multiple comparisons test. *$p < 0.05$; **$p < 0.01$; ***$p < 0.001$; ****$p < 0.0001$.

Table S1. Basic information for volunteers of centenarians and controls, Related to Figure 1, 2, and 4

Random#	Gender	Age	Ethnicity	Group	ATACseq	ATAC.QC	RNAseq	RNA.QC	Data.availability
C1	Male	102	Chinese	Centenarians	Y	PASSED	N		CA
C2	Female	104	Chinese	Centenarians	Y	PASSED	Y	PASSED	CA+GE
C3	Male	99	Chinese	Centenarians	Y	FAILED	Y	PASSED	GE
C4	Female	99	Chinese	Centenarians	Y	PASSED	N		CA
C5	Female	101	Chinese	Centenarians	Y	PASSED	Y	PASSED	CA+GE
C6	Male	100	Chinese	Centenarians	Y	PASSED	Y	PASSED	CA+GE
C7	Female	100	Chinese	Centenarians	Y	PASSED	N		CA
C8	Male	101	Chinese	Centenarians	Y	FAILED	Y	PASSED	GE
C9	Male	102	Chinese	Centenarians	Y	PASSED	Y	PASSED	CA+GE
C10	Male	102	Chinese	Centenarians	Y	PASSED	N		CA
C11	Male	101	Chinese	Centenarians	Y	PASSED	Y	PASSED	CA+GE
C13	Female	102	Chinese	Centenarians	Y	PASSED	Y	PASSED	CA+GE
C14	Female	100	Chinese	Centenarians	Y	PASSED	Y	PASSED	CA+GE
C15	Male	101	Chinese	Centenarians	Y	PASSED	Y	PASSED	CA+GE
C16	Female	104	Chinese	Centenarians	Y	PASSED	Y	PASSED	CA+GE+qPCR
C17	Female	104	Chinese	Centenarians	Y	PASSED	Y	PASSED	CA+GE
C18	Male	103	Chinese	Centenarians	Y	PASSED	Y	PASSED	CA+GE
C19	Male	100	Chinese	Centenarians	Y	PASSED	Y	PASSED	CA+GE
C20	Male	104	Chinese	Centenarians	Y	PASSED	Y	PASSED	CA+GE
C21	Female	100	Chinese	Centenarians	Y	PASSED	Y	PASSED	CA+GE+qPCR
C22	Female	104	Chinese	Centenarians	Y	PASSED	Y	PASSED	CA+GE+qPCR
C23	Female	102	Chinese	Centenarians	Y	PASSED	Y	PASSED	CA+GE
C24	Female	106	Chinese	Centenarians	Y	PASSED	Y	PASSED	CA+GE
C25	Female	100	Chinese	Centenarians	Y	PASSED	Y	PASSED	CA+GE
C26	Female	100	Chinese	Centenarians	Y	PASSED	Y	PASSED	CA+GE
C27	Female	101	Chinese	Centenarians	Y	PASSED	Y	PASSED	CA+GE
C28	Female	103	Chinese	Centenarians	Y	PASSED	Y	PASSED	CA+GE
C29	Female	101	Chinese	Centenarians	Y	PASSED	Y	PASSED	CA+GE
C30	Female	100	Chinese	Centenarians	Y	PASSED	Y	PASSED	CA+GE
C31	Female	103	Chinese	Centenarians	Y	PASSED	Y	PASSED	CA+GE
C32	Female	100	Chinese	Centenarians	Y	PASSED	Y	PASSED	CA+GE
C33	Female	101	Chinese	Centenarians	Y	FAILED	Y	PASSED	GE
NC1	Female	55	Chinese	Controls	Y	PASSED	N		CA
NC2	Male	66	Chinese	Controls	Y	FAILED	N		
NC3	Female	64	Chinese	Controls	Y	PASSED	Y	PASSED	CA+GE
NC4	Male	69	Chinese	Controls	Y	FAILED	Y	PASSED	GE
NC5	Female	53	Chinese	Controls	Y	FAILED	Y	PASSED	GE
NC6	Female	55	Chinese	Controls	N		Y	PASSED	GE
NC7	Female	59	Chinese	Controls	Y	FAILED	Y	PASSED	GE
NC8	Female	65	Chinese	Controls	Y	FAILED	Y	PASSED	GE
NC9	Female	68	Chinese	Controls	Y	FAILED	Y	PASSED	GE
NC10	Female	60	Chinese	Controls	Y	PASSED	Y	PASSED	CA+GE
NC11	Female	65	Chinese	Controls	Y	PASSED	Y	PASSED	CA+GE
NC12	Female	56	Chinese	Controls	Y	PASSED	Y	PASSED	CA+GE
NC13	Male	56	Chinese	Controls	Y	PASSED	Y	PASSED	CA+GE
NC14	Female	67	Chinese	Controls	N		Y	PASSED	GE
NC15	Female	55	Chinese	Controls	Y	PASSED	Y	PASSED	CA+GE
NC16	Female	53	Chinese	Controls	N		Y	PASSED	GE
NC17	Male	65	Chinese	Controls	Y	PASSED	Y	PASSED	CA+GE
NC18	Male	59	Chinese	Controls	N		Y	PASSED	GE
NC19	Female	54	Chinese	Controls	Y	FAILED	Y	PASSED	GE+qPCR
NC20	Male	68	Chinese	Controls	Y	PASSED	Y	PASSED	CA+GE
NC21	Female	62	Chinese	Controls	Y	PASSED	Y	PASSED	CA+GE+qPCR
NC22	Female	56	Chinese	Controls	Y	PASSED	Y	PASSED	CA+GE
NC23	Male	59	Chinese	Controls	N		Y	PASSED	GE+qPCR
NC24	Female	67	Chinese	Controls	Y	PASSED	N		CA+qPCR
NC26	Female	67	Chinese	Controls	N		Y	PASSED	GE
NC28	Female	65	Chinese	Controls	N		Y	PASSED	GE
NC29	Female	54	Chinese	Controls	N		Y	PASSED	GE
NC31	Male	69	Chinese	Controls	N		Y	PASSED	GE
NC32	Female	70	Chinese	Controls	N		Y	PASSED	GE
NC33	Female	59	Chinese	Controls	N		Y	PASSED	GE

NC34	Male	66	Chinese	Controls	Y	PASSED	Y	PASSED	CA+GE
NC35	Male	70	Chinese	Controls	N		Y	PASSED	GE
NC37	Female	60	Chinese	Controls	Y	PASSED	Y	PASSED	CA+GE
NC38	Female	66	Chinese	Controls	Y	FAILED	Y	PASSED	GE
NC39	Male	67	Chinese	Controls	Y	PASSED	N		CA
NC40	Female	72	Chinese	Controls	Y	PASSED	Y	PASSED	CA+GE
NC41	Male	62	Chinese	Controls	Y	PASSED	Y	PASSED	CA+GE
NC42	Female	67	Chinese	Controls	Y	PASSED	Y	PASSED	CA+GE
NC44	Male	66	Chinese	Controls	Y	PASSED	Y	PASSED	CA+GE
NC45	Female	67	Chinese	Controls	Y	PASSED	N		CA
NC46	Male	73	Chinese	Controls	Y	PASSED	N		CA
NC47	Female	69	Chinese	Controls	Y	FAILED	N		
NC48	Male	72	Chinese	Controls	Y	FAILED	N		
NC49	Female	69	Chinese	Controls	Y	FAILED	N		

CA: Chromatin accessibility; GE: Gene expression

Table S2. Primers and sequences used in this study, related to STAR Methods

Oligonucleotide	Sequence (5′ to 3′)	Purpose
pET20b-ERG-F	CAGCAAATGGGTCGCGGATCC ATGGCCAGCACTATTAAG	Plasmids constructs
pET20b-ERG-R	ACGGAGCTCGAATTCGGATCC TTAGTAGTAAGTGCCCAG	Plasmids constructs
pLV-ERG-F	GACGGTACCGCGGGCCCGGGATCC ATGGCCAGCACTATTAAG	Plasmids constructs
pLV-ERG-R	CAGTTATCTAGATCCGGTGGATCCTT AGTAGTAAGTGCCCAG	Plasmids constructs
GFP-ERG-F	TACAAGGAATTCGAGCTCCGTCGACA AATGGCCAGCACTATTAAG	Plasmids constructs
GFP-ERG-R	TCGAGTGCGGCCGCAAGCTTAGTAGTAAGTGCCCAG	Plasmids constructs
D1-F	TACAAGGAATTCGAGCTCCGTCGACAA ATGGCCAGCACTATTAAG	Plasmids constructs
D1-R	TCGAGTGCGGCCGCAAGCTTAGTAGTAAGTGCCCAG	Plasmids constructs
D2-F	TACAAGGAATTCGAGCTCCGTCGACAA ATGGCCAGCACTATTAAG	Plasmids constructs
D2-R	TCGAGTGCGGCCGCAAGCTTAGTAGTAAGTGCCCAG	Plasmids constructs
D3-F	TACAAGGAATTCGAGCTCCGTCGAC AAATGGCCAGCACTATTAAG	Plasmids constructs
D3-R	TCGAGTGCGGCCGCAAGC TTAGAGGGCCTGGGC	Plasmids constructs
D13-F	GAACAGATTGGTGGTCTCGAGATGGCCAGCACTATTAAG	Plasmids constructs
D13-R	GTGGTGGTGGTGGTGGTGCTCGAG TTAGAGGGCCTGGGC	Plasmids constructs
D12-F	AGAGAACAGATTGGTGGTCTCGAG ATGGCCAGCACTATTAAG	Plasmids constructs
D12-R	TCAGTGGTGGTGGTGGTGGTGCTCGAG TTAGTAGTAAGTGCCCAG	Plasmids constructs
D23-F	AGAGAACAGATTGGTGGT CTCGAG ATGGCCAGCACTATTAAG	Plasmids constructs
D23-F	TCAGTGGTGGTGGTGGTGGTGCTCGAG TTAGAGGGCCTGGGCGAT	Plasmids constructs
Promoter-F	AGATCTGCGATCTAAGTAGCATGTGGCCCCAGCACA	Plasmids constructs
Promoter-R	GTACCGGAATGCCAAGCTGAGCAGCGCCACTCCTGC	Plasmids constructs
ERG-F	TATGGCCTTCCAGACGTCAAC	RT-qPCR; ddPCR
ERF-R	GTCAAATGTGGAAGAGGAGTCTCT	RT-qPCR; ddPCR
Si-ERG-SS	GACGUCAACAUCUUGUUAUTT	Knock down
Si-ERG-AS	AUAACAAGAUGUUGACGUCTT	Knock down
P16-F	GGGTTTTCGTGGTTCACATCC	RT-qPCR
P16-R	CTAGACGCTGGCTCCTCAGTA	RT-qPCR
P21-F	TGTCCGTCAGAACCCATGC	RT-qPCR
P21-R	AAAGTCGAAGTTCCATCGCTC	RT-qPCR
IL6-F	AAG CCA GAGC TGT GCA GAT GAG TA	RT-qPCR
IL6-R	TGT CCT GCA GCC ACT GGT TC	RT-qPCR
IL8-F	ACC GGA AGG AAC CAT CTC AC	RT-qPCR
IL8-R	AAA CTG CAC CTT CAC ACA GAG	RT-qPCR
MMP1-F	CAT CGT GTT GCA GCT CAT GA	RT-qPCR
MMP1-R	ATG GGC TGG ACA GGA TTT TG	RT-qPCR
TGFB3-F	ACTTGCACCACCTTGGACTTC	RT-qPCR
TGFB3-R	GGTCATCACCGTTGGCTCA	RT-qPCR
KRAS-F	ACAGAGAGTGGAGGATGCTTT	RT-qPCR
KRAS-R	TTTCACACAGCCAGGAGTCTT	RT-qPCR
RBL2-F	CCACCCCTCAGATCCAGCA	RT-qPCR
RBL2-R	CGTGTAGCTTTCGCTCATGC	RT-qPCR
HIPK3-F	TCACAAGTCTTGGTCTACCCA	RT-qPCR
HIPK3-R	CACATAGGTCCGTGGATAGTTTC	RT-qPCR
qP-actin-F	CATGTACGTTGCTATCCAGGC	RT-qPCR
qP-actin-R	CTCCTTAATGTCACGCACGAT	RT-qPCR
ERG-ALF	ACGGACCGGAAATCCGGTT	EMSA
ERG-ALR	AACCGGATTTCCGGTCCGT	EMSA
ChIP-F	CTCTTTCTTCCTCCGGTGCTG	ChIP-qPRC
ChIP-R	TCCCCTTGCCTGGAAAGATAC	ChIP-qPRC
